# Long‐Term Stability of Aboveground Biomass and Productivity Without Anthropogenic Management in Subtropical Coniferous Forests: Insights From the Ecosystem Demography Model

**DOI:** 10.1002/ece3.74660

**Published:** 2026-11-13

**Authors:** Jiajia Wang, Yu Zhu, Shuguang Liu, Quanlin Li, Zhao Wang, Meifang Zhao, Liangjun Zhu

**Affiliations:** ^1^ School of Ecology and Environment Central South University of Forestry and Technology Changsha China; ^2^ National Engineering Laboratory for Applied Technology of Forestry & Ecology in South China Central South University of Forestry and Technology Changsha China; ^3^ Technology Innovation Center for Ecological Conservation and Restoration in Dongting Lake Basin Ministry of Natural Resources Changsha China; ^4^ School of Ecology Hainan University Haikou China; ^5^ School of Ecology Northeast Forestry University Harbin China

**Keywords:** aboveground biomass, drought stress, Ecosystem Demography model, forest succession, subtropical coniferous forest, temporal stability

## Abstract

Understanding the long‐term stability of forest ecosystems is essential for predicting the sustainability of forest carbon sequestration under climate change. However, the temporal dynamics of stability in subtropical coniferous forests during natural succession remain poorly understood. Filling this knowledge gap is necessary to establish a baseline for disentangling the combined effects of anthropogenic and natural disturbances. Using the process‐based Ecosystem Demography model (ED‐2.2), we simulated multi‐decadal dynamics of aboveground biomass (AGB), productivity, and their stability in coniferous forests of subtropical China under a scenario excluding anthropogenic management. Our results indicated that the temporal stability of simulated forest productivity was generally higher in the early development period (i.e., 1–50 simulation years, 0.647 ± 0.574) than in the late development period (i.e., 51–97 simulation years, −0.061 ± 0.168), with negative stability values reflecting periods of net AGB loss. Forest plots with lower stability of simulated productivity (−0.187 to −0.131) also exhibited reduced stability of simulated AGB (1.812–3.024) in the late period. It was likely associated with seasonal precipitation stress between summer and autumn according to model simulations and subtropical climate features. Seasonal precipitation stress may trigger a cascade of effects, ranging from reduced water availability and leaf photosynthesis to elevated tree mortality risk. These changes ultimately alter forest composition and structure, destabilizing annual productivity. Our findings highlight that precipitation played a key role in shaping the long‐term stability of productivity in subtropical coniferous forests.

## Graphical abstract

In certain plots, AGB generally accumulated over time and eventually approached a dynamic equilibrium. In contrast, in other plots, total AGB underwent a sharp decline at a tipping point in the late development stage, coinciding with the AGB of shade‐tolerant broadleaves surpassing that of light‐demanding conifers.

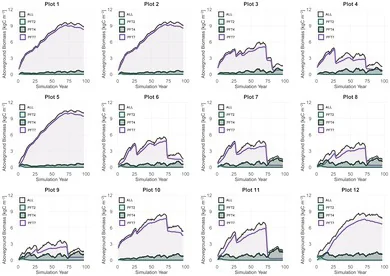

## 1 Introduction

Forests play an irreplaceable role in regulating the global carbon balance, mitigating the rise of atmospheric greenhouse gas concentrations (e.g., CO_2_), and responding to climate change (Fang et al. 2001; Mo et al. 2023; Pan et al. 2024; Piao, He, et al. 2022). The capacity of forest carbon sequestration, along with the associated stability, temporal variability, and sustainability, represented a critical scientific challenge in achieving carbon neutrality goals (Piao, Yue, et al. 2022; Wang et al. 2021). The temporal stability of forest carbon sequestration is typically quantified as the mean biomass and/or productivity divided by their standard deviation over time (Jucker et al. 2014; Tilman 1999). Means approach zero typically indicate approximate growth‐mortality balance in biomass and/or productivity. During long‐term development, forests typically experience extreme climate events (e.g., severe droughts) occurring only once in several decades (Ding et al. 2024; Trenberth et al. 2013). Therefore, understanding the long‐term stability of forest carbon sequestration is essential for ensuring the sustainability of forest carbon sequestration in a changing climate.

Forest stability of carbon sequestration over time is modulated by a complex set of interacting factors, including biotic community characteristics (e.g., diversity, age, and structure) as well as abiotic disturbances (e.g., harvest, fire, and drought) (Qiao et al. 2023a, 2023b; Roces‐Díaz et al. 2025; Shan et al. 2025; Wales et al. 2020). Among these, tree diversity plays a key role in regulating the temporal stability of forest biomass and productivity. In this context, PFT richness has been suggested as a useful proxy for one functional dimension of tree diversity (Gillison 2002; Gillison et al. 2013; Liu et al. 2013). Such diversity–stability relationships have been consistently documented across boreal, temperate, and tropical forests using forest inventories, field experiments, and virtual simulations. (Ding et al. 2024; Jucker et al. 2014; Morin et al. 2014; Ouyang et al. 2020; Qiao et al. 2023a, 2023b; Schnabel et al. 2021, 2019). In addition, anthropogenic and natural disturbances can either stabilize or destabilize forest ecosystems during development. For example, clear‐cut harvesting and subsequent fires has been shown to exhibit higher temporal stability of stand production compared to clear‐cut harvesting only in temperate forests (Wales et al. 2020). In contrast, selective logging has been shown to reduce aggregate stability and compositional stability across the local (*α*) and larger (*γ*) spatial scales (Qiao et al. 2023a, 2023b). However, how the temporal stability of forest evolves over the long‐term development excluding anthropogenic management (e.g., selective logging) remains poorly understood. Quantifying stability of carbon sequestration under natural succession is crucial, as it establishes a fundamental baseline for discerning the complex, combined effects of anthropogenic management and natural disturbances on forest stability of carbon sequestration.

In recent years, the process–based forest models have become useful tool in exploring forest structure, function, and their temporal stability through virtual experiment simulations (i.e., in silico experiments) (Levine et al. 2016; Longo et al. 2025; Morin et al. 2014; Schmid et al. 2021). Morin et al. (2014) employed forest succession model (i.e., ForClim) to test forests diversity–stability relationship by carrying out virtual biodiversity experiment simulations. Similarly, Longo et al. (2025) used cohort‐based model (i.e., ED‐2.2) to investigate how forest structure responds to extreme climate events through switching on or off vegetation dynamics. Process‐based forest vegetation demographic models (e.g., ED‐2.2) offer three key advantages for simulating long‐term forest stability of carbon sequestration. First, they mechanistically represent carbon and water cycling, thereby clarifying underlying causal processes (Bonan 2019). Second, they explicitly capture both horizontal and vertical heterogeneity within ecosystems, making it possible to track long–term changes in forest structure and composition (Longo et al. 2019; Medvigy et al. 2009; Moorcroft et al. 2001). Third, they allow us to exclude anthropogenic management, facilitating the study of how forest productivity stabilizes under purely natural disturbance regimes (Longo et al. 2019).

Coniferous forests play a critical role in the global forest carbon sink, characterized by high productivity and substantial carbon storage capacity across major subtropical regions (Liu, Feng, et al. 2024; Liu, Lie, et al. 2024; Yan et al. 2023; Yu et al. 2014). For instance, actively managed pine stands serve as persistent carbon sinks in the subtropical United States (Ameray et al. 2021; Coulston et al. 2015), while large‐scale afforestation projects in China involving species such as *Cunninghamia lanceolata* and *Pinus massoniana* have significantly enhanced regional carbon stocks (Yu et al. 2024). However, in recent decades, subtropical coniferous forests have faced increasing threats from extreme climate events. Evidence from tree‐ring data, process‐based models, and remote‐sensing observations consistently indicated that drought, particularly seasonal drought, has significantly constrained tree growth and biomass productivity in these ecosystems (Yan et al. 2023; Yuan et al. 2016; Zhang et al. 2023). Therefore, understanding the long‐term stability of biomass production in subtropical coniferous forests under climate change is essential to sustaining their carbon sequestration capacity and associated ecosystem services.

We simulated multi‐decadal dynamics of aboveground biomass, productivity, and their stability in subtropical coniferous forests of China using the process‐based Ecosystem Demography model (ED‐2.2), under a scenario excluding anthropogenic management. This study intends to address the following questions:

1. How does the temporal stability of carbon sequestration vary during long‐term development in subtropical coniferous forests? It was hypothesized that there was a general decline in the temporal stability of annual productivity over time without anthropogenic management. According to previous studies on the relationships of tree diversity and stand structure with ecosystem stability, we expected that productivity stability would be positively associated with PFT richness and stand structural complexity during stand development (Ding et al. 2024; Gillison 2002; Gillison et al. 2013; Liu et al. 2013; Liu, Feng, et al. 2024; Ouyang et al. 2020; Roces‐Díaz et al. 2025; Shan et al. 2025).
2. What is the key meteorological factor governing the long‐term stability of carbon sequestration in subtropical coniferous forests? It was hypothesized that precipitation would be a stronger indicator of stability than air temperature or radiation. Because previous studies have indicated that evergreen coniferous forests typically exhibited lower resistance to severe drought compared to evergreen broadleaved forests, reflecting the higher sensitivity of conifers to drought, particularly in subtropical China (Huang and Xia 2019; Yan et al. 2023; Zhang et al. 2023).

## 2 Materials and Methods

### 2.1 Sites Description

Subtropical coniferous forests, dominated by Chinese fir ( *Cunninghamia lanceolata* ) and Masson pine ( *Pinus massoniana* ), represent two of the most widespread forest types in subtropical China. The study area is situated in southwestern Hunan Province, a core distribution zone for such forests. These ecosystems provide essential ecological services, including timber production, biodiversity conservation, carbon sequestration, water regulation, and soil fertility maintenance. Furthermore, they play a vital role in mitigating climate change and safeguarding regional ecological security. The selected forest inventory plots (see Section 2.2.1) span a range of climatic conditions. According to the MERRA‐2 reanalysis product (see Section 2.2.2), mean annual temperature ranges from 15.8°C to 17.5°C, with a mean of 16.4°C; total annual precipitation ranges from 1259 to 1522 mm, with a mean of 1347 mm.

### 2.2 Data Collection

#### 2.2.1 Forest Inventory Data

We selected 12 forest permanent plots of subtropical coniferous forests (i.e., *C. lanceolata* forests and *P. massoniana* forests) from the fifth (2004) National Forest Inventory of China in Hunan Province (Table S1). Each plot of 666.7 m^2^ contained stand‐level factors and tree‐level attributes (e.g., DBH and species). Most plots represented young to mid‐aged stands (< 20 years) in 2004. Forest inventory data in 2004 were used to initialize vegetation status for the ED‐2.2 model for each plot. Allometric growth data of trees are sourced from the Tallo database (Jucker et al. 2022).

#### 2.2.2 Atmospheric and Edaphic Data

We used hourly averages (1980–2023) from the Modern‐Era Retrospective Analysis for Research and Applications, Version 2 (MERRA‐2) reanalysis data (e.g., temperature, specific humidity, pressure, precipitation rate, incoming solar irradiance, and thermal irradiance) as meteorological forcing to drive the ED‐2.2 model simulation (Gelaro et al. 2017; GMAO 2015a, 2015b, 2015c, 2015d). For future projections (2024–2100), we assumed that the model was driven by sequentially recycling MERRA‐2 reanalysis from historical period (1980–2023). The objective of this study is to analyze stability under stationary climate variability (via repeated MERRA‐2 historical climate forcing), rather than climate change projection. Soil textures were derived from SoilGrids 2.0 (Hengl et al. 2017; Poggio et al. 2021).

#### 2.2.3 PFT‐Specific Parameters

The PFT‐dependent parameters (e.g., SLA, specific leaf area; V_cmax_, maximum Rubisco carboxylation rate; WD, wood density; b1Ht and b2Ht, DBH‐height allometric coefficients) were obtained from the literatures (Table S5) (Chave et al. 2009; Dong 2015; Dong et al. 2015; Kattge et al. 2020; LeBauer et al. 2018; Li and Xiang 2011; Li et al. 2007, 2018; Li 2016, 2019; Paine et al. 2015; Prentice et al. 2011; Wang et al. 2022, 2018; Wang 2010; Wu et al. 2021; Xie et al. 2022; Xu, Wang, Prentice, Harrison, Wang, and Sun 2021; Xu, Wang, Prentice, Harrison, and Wright 2021; Ye 2019; Zhou 2013; Zhu et al. 2022).

### 2.3 Ecosystem Demography Model

#### 2.3.1 Model Description

The latest release version of ED‐2.2 (Ecosystem Demography, Version 2.2) is a cohort‐based and process‐based vegetation model that couples biophysics, demographic dynamics, and soil biogeochemistry (https://github.com/EDmodel/ED2) (Longo et al. 2019; Medvigy et al. 2009; Moorcroft et al. 2001). The ED‐2.2 model accounts for the horizontal and vertical heterogeneity of the ecosystem through its cohort/patch structure, capturing size‐structured competition and multi‐timescale processes: sub‐daily canopy biophysics and thermodynamics, daily phenology, and monthly cohort demography (Longo et al. 2019). Annual productivity (ΔAGB) is not prescribed by an empirical function; rather, it emerges from the coupled interactions of leaf‐level photosynthesis (Farquhar et al. 1980), stomatal conductance (Leuning 1995), respiration, canopy radiation transfer, and demographic processes (e.g., growth, mortality, recruitment). The ED series models has been widely applied at both site and region scales to quantify the impacts of forest management and climate variability on ecosystem carbon dynamics and to support carbon mapping (Cheng et al. 2024; Longo et al. 2025; Ma et al. 2022; Xu, Konings, et al. 2021).

#### 2.3.2 Model Configuration and Validation

The plant functional type (PFT) is a group of plant species that respond similarly to environmental conditions. *C. lanceolata* and *P. massoniana* can be identified as the subtropical light‐demanding coniferous plant functional type (PFT7) in the ED‐2.2 model configuration. Subtropical evergreen broadleaved trees are divided into light‐demanding evergreen broadleaved species (PFT2), for example, *Camphora officinarum*, and shade‐tolerant evergreen broadleaved species (PFT4), for example, *Quercus glauca* .

The performance of the model was evaluated by using the observed aboveground biomass of different age classes of subtropical coniferous forests in Hunan Province of China (Wang et al. 2024). The model error was assessed and the simulation precision was validated using the mean absolute error (MAE), root mean square error (RMSE), and the weighted least squares method (Φ) (Liu et al. 2008) as evaluation metrics. For details regarding model structural optimization, parameter calibration, and validation, please refer to the Supporting Information (S2 and S3, Tables S2–S5).

#### 2.3.3 Model Simulations and Sensitivity Analyses

Previous studies have found that anthropogenic management can strongly alter forest stability (Qiao et al. 2023a; Wales et al. 2020). We employed the commonly used virtual simulation experiments in recent years (Levine et al. 2016; Longo et al. 2025; Morin et al. 2014), assuming no anthropogenic management throughout the simulation period, to simulate the long‐term stability of aboveground biomass and productivity of subtropical coniferous forests under natural disturbance conditions only. This study aimed to investigate the forest stability under a baseline scenario without anthropogenic management through virtual simulations. Quantifying forest stability under natural succession is crucial for establishing a baseline to understand the compound effects of anthropogenic disturbances on forest ecosystems.

Annual aboveground biomass (AGB, kg C m^−2^) was extracted from the model simulations. The annual net change in AGB, termed “productivity (kg C m^−2^ year^−1^)” was computed as:

(1) $$\text{Productivity}={\mathrm{AGB}}_{t+1}-{\mathrm{AGB}}_{t}$$

where AGB_*t*+1_ and AGB_*t*_ indicate the AGB values at time steps *t* (current year) and *t* + 1 (subsequent year), respectively.

The “productivity” reported here is defined as the annual net change in AGB, that is, the net stock change of aboveground carbon between two consecutive time steps, rather than traditional NPP. As a stock‐change metric, “productivity” integrates multiple processes represented in ED‐2.2 (e.g., growth, mortality) and therefore can be negative when AGB losses (e.g., mortality) exceed gains from growth.

Intermediate variables included monthly gross primary production (GPP), water stress factor, available water, leaf transpiration, leaf temperature, and absorbed photosynthetically active radiation by leaves. Following previous ED modeling studies (Albani et al. 2006; Feng et al. 2018; Medvigy et al. 2009), we selected SLA and V_cmax_ as key ecophysiological parameters and performed a sensitivity analysis to test the robustness of our simulation results. Both parameters directly regulated leaf photosynthetic capacity and carbon uptake (Bonan 2019; Farquhar et al. 1980; von Caemmerer et al. 2025).

The temporal stability metric of each plot over a period is defined as the ratio of the mean to the standard deviation of aboveground biomass and productivity values during that period (Jucker et al. 2014; Wang and Loreau 2016):

(2) $$\text{Stability}=\frac{\text{Mean}}{\mathrm{SD}}$$

where mean and SD represented the mean and standard deviation, respectively, of the annual‐scale forest aboveground biomass or productivity during that period. We also calculated absolute temporal stability and detrended stability. Detrended stability: we replaced the SD of aboveground biomass/annual productivity with the SD of the residuals of the linear regression between aboveground biomass/annual productivity and simulation year (Shan et al. 2025; Tilman et al. 2006).

All the statistical computing and graphics were implemented in R v.4.1.1 (R_Core_Team 2021).

## 3 Results

### 3.1 Stability of Forest Aboveground Biomass and Productivity

The temporal dynamics of simulated forest composition, structure, and aboveground biomass (AGB) revealed two distinct development trajectories. In Group 1 plots (e.g., Plots 1, 2, 5, and 12), simulated AGB generally accumulated over time and eventually approached a dynamic equilibrium (Figures 1 and 2). Light‐demanding conifers consistently dominated the canopy in terms of AGB contribution. Throughout most of the simulation, large trees predominated in the canopy, except during the early development period (i.e., 1–50 simulation years) when middle and small trees were dominant (Figures 1 and 2). By the end of the simulation, the canopy was primarily composed of large conifers (Figures 1 and 2). These forest plots maintained high stability of productivity and exhibited increasing stability of AGB over time (Tables 1, 2, 3 and Tables S6–S10).

**FIGURE 1:**
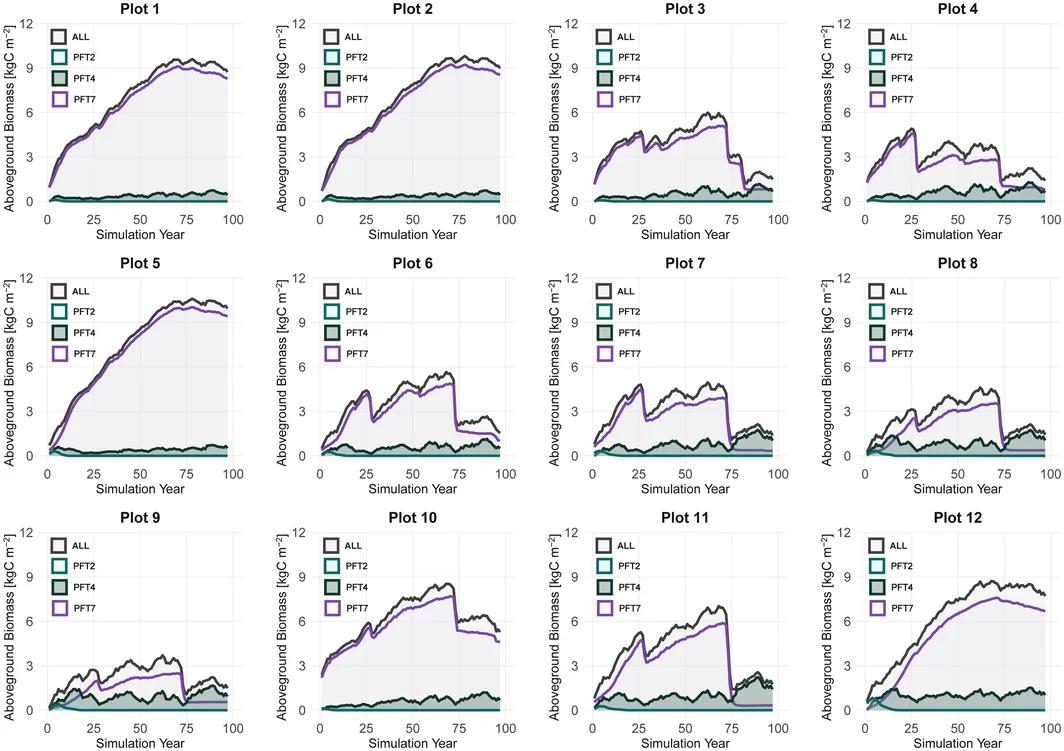
Long‐term trajectory of simulated forest composition and aboveground biomass (kg C m^−2^) from ED‐2.2 model simulation. Gray indicates total trees; purple indicates light‐demanding coniferous trees (PFT7); dark green indicates shade‐tolerant evergreen broadleaved trees (PFT4); light green indicates light‐demanding evergreen broadleaved trees (PFT2).

**FIGURE 2:**
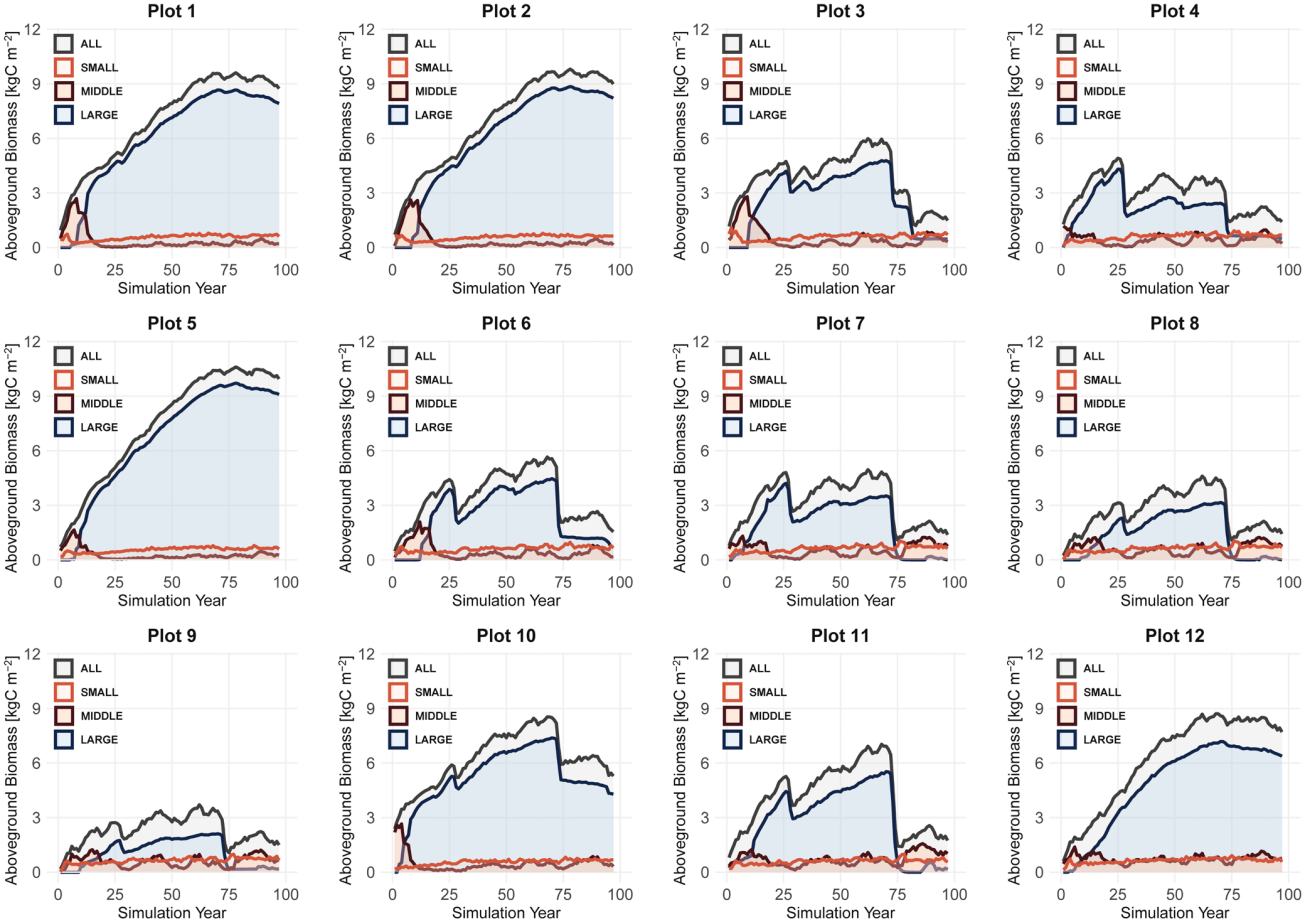
Long‐term trajectory of simulated forest structure and aboveground biomass (kg C m^−2^) from ED‐2.2 model simulation. Gray indicates total trees; blue indicates large‐sized trees (DBH > 30 cm); dark red indicates medium‐sized trees (10 cm < DBH ≤ 30 cm); light red indicates small‐sized trees (DBH ≤ 10 cm).

**TABLE 1:** Temporal stability of simulated aboveground biomass and forest productivity during stand development (i.e., 1–97 simulation years).

Plot		Aboveground biomass			Forest productivity		
		Mean	SD	Stability	Mean	SD	Stability
1		7.118	2.345	3.035	0.081	0.130	0.626
2		7.102	2.486	2.857	0.086	0.128	0.675
3		3.827	1.358	2.817	0.003	0.308	0.011
4		3.044	0.955	3.187	0.001	0.316	0.005
5		7.574	2.919	2.595	0.096	0.114	0.844
6		3.483	1.336	2.606	0.011	0.350	0.032
7		3.209	1.226	2.617	0.006	0.320	0.020
8		2.695	1.101	2.448	0.012	0.271	0.045
9		2.328	0.751	3.101	0.014	0.219	0.063
10		6.230	1.457	4.277	0.030	0.239	0.127
11		4.104	1.801	2.279	0.010	0.379	0.026
12		6.306	2.407	2.620	0.074	0.153	0.483
Total	Mean	4.752	1.679	2.870	0.035	0.244	0.246
	SD	1.953	0.699	0.519	0.037	0.094	0.315

*Note:* Temporal stability was defined as mean/SD.

**TABLE 2:** Temporal stability of simulated aboveground biomass and forest productivity during early development period (i.e., 1–50 simulation years).

Plot		Aboveground biomass			Forest productivity		
		Mean	SD	Stability	Mean	SD	Stability
1		5.193	1.728	3.005	0.142	0.119	1.198
2		5.042	1.774	2.842	0.146	0.116	1.253
3		3.960	0.752	5.266	0.072	0.218	0.332
4		3.474	0.806	4.311	0.046	0.353	0.131
5		5.194	2.190	2.372	0.160	0.083	1.918
6		3.368	1.167	2.886	0.087	0.258	0.336
7		3.436	0.964	3.563	0.064	0.283	0.225
8		2.587	0.933	2.773	0.066	0.202	0.326
9		2.248	0.711	3.163	0.053	0.185	0.285
10		5.472	1.373	3.985	0.102	0.164	0.623
11		4.096	1.271	3.224	0.096	0.237	0.405
12		4.444	2.034	2.185	0.139	0.133	1.039
Total	Mean	4.043	1.309	3.298	0.124	0.196	0.647
	SD	1.059	0.514	0.869	0.098	0.079	0.574

*Note:* Temporal stability was defined as mean/SD.

**TABLE 3:** Temporal stability of simulated aboveground biomass and forest productivity during late development period (i.e., 51–97 simulation years).

Plot		Aboveground biomass			Forest productivity		
		Mean	SD	Stability	Mean	SD	Stability
1		9.148	0.398	23.012	0.015	0.109	0.136
2		9.276	0.464	19.979	0.022	0.109	0.198
3		3.663	1.795	2.040	**−**0.073	0.372	**−**0.196
4		2.578	0.899	2.869	**−**0.044	0.271	**−**0.161
5		10.085	0.479	21.062	0.027	0.103	0.261
6		3.579	1.504	2.380	**−**0.069	0.418	**−**0.164
7		2.947	1.427	2.069	**−**0.059	0.349	**−**0.170
8		2.789	1.260	2.214	**−**0.050	0.322	**−**0.155
9		2.399	0.793	3.024	**−**0.032	0.244	**−**0.131
10		7.006	1.088	6.441	**−**0.051	0.280	**−**0.181
11		4.075	2.250	1.812	**−**0.088	0.472	**−**0.187
12		8.262	0.318	25.958	0.003	0.144	0.022
Total	Mean	5.484	1.056	9.405	**−**0.033	0.266	**−**0.061
	SD	3.009	0.611	9.844	0.040	0.127	0.168

*Note:* Temporal stability was defined as mean/SD.

In Group 2 plots (e.g., Plots 7, 8, 9, and 11) exhibited a different trajectory: simulated total AGB underwent a sharp decline at a tipping point in the late development stage, coinciding with the AGB of shade‐tolerant broadleaves surpassing that of light‐demanding conifers (Figures 1 and 2). Simultaneously, middle and small trees exceeded large trees in AGB. By the end of the simulation, the canopy was largely dominated by middle and small broadleaved trees (Figures 1 and 2). These forest plots, with consistently low stability of productivity, showed a trend of declining stability of AGB (Tables 1, 2, 3 and Tables S6–S10).

The stability of simulated forest productivity was generally higher during the early development period (0.647 ± 0.574) of stand development than during the late development period (i.e., 51–97 simulation years) (−0.061 ± 0.168) for all forest plots (Tables 1, 2, 3 and Tables S6–S10). Forest plots with lower stability of productivity (−0.187 to −0.131) also exhibited reduced stability of AGB (1.812–3.024) in the late period (Table 3). Sensitivity analyses were conducted by varying SLA and V_cmax_ by ±10% and ±20%. The results indicated that simulated AGB was highly sensitive to changes in these parameters (Table S11, Figures S1 and S2). Nevertheless, such parameter variations did not alter the overall temporal trajectory of total AGB simulated using the baseline parameters. Sensitivity analyses supported the robustness of simulation results.

During late development period, these systems are not approaching equilibrium nor experiencing sustained decline, but are in a successional dynamic adjustment process (e.g., Plots 7, 8, 9, and 11). Negative mean productivity indicates a temporary period of net biomass loss, neither sustained loss nor model equilibrium dynamics. This transient decline in biomass is caused by a short‐term increase in tree mortality, and productivity will gradually recover as the ecosystem adjusts to a new dynamic equilibrium.

Regardless of which stability metric we used (i.e., temporal stability, absolute temporal stability, or detrended stability), the results were largely consistent: the temporal stability of annual productivity was generally higher in early period than in late period (Tables 1, 2, 3 and Tables S12–S17). Forest plots with lower stability of annual productivity also exhibited decreased stability of AGB in late period (Tables 1, 2, 3 and Tables S12–S17).

### 3.2 Variability for Meteorological and Intermediate Variables

The model captured distinct patterns of interannual variability between two groups of plots (Group 1: Plots 1, 2, 5, and 12 vs. Group 2: Plots 7–9, and 11). In Group 1, interannual variability was consistently low across all months for simulated GPP, water stress factor (weighting factor for open and closed stomata), and leaf transpiration (Figures 3 and 4, and Figure S3). For available water, interannual variability was generally lower during summer and autumn compared to spring and winter (Figure 5). In contrast, Group 2 plots exhibited a different pattern. These plots showed elevated interannual variability during the summer–autumn transition for simulated GPP, water stress factor, and leaf transpiration (Figures 3 and 4, and Figure S3). However, the interannual variability of available water during this transition period was comparable to that in other months (Figure 5).

**FIGURE 3:**
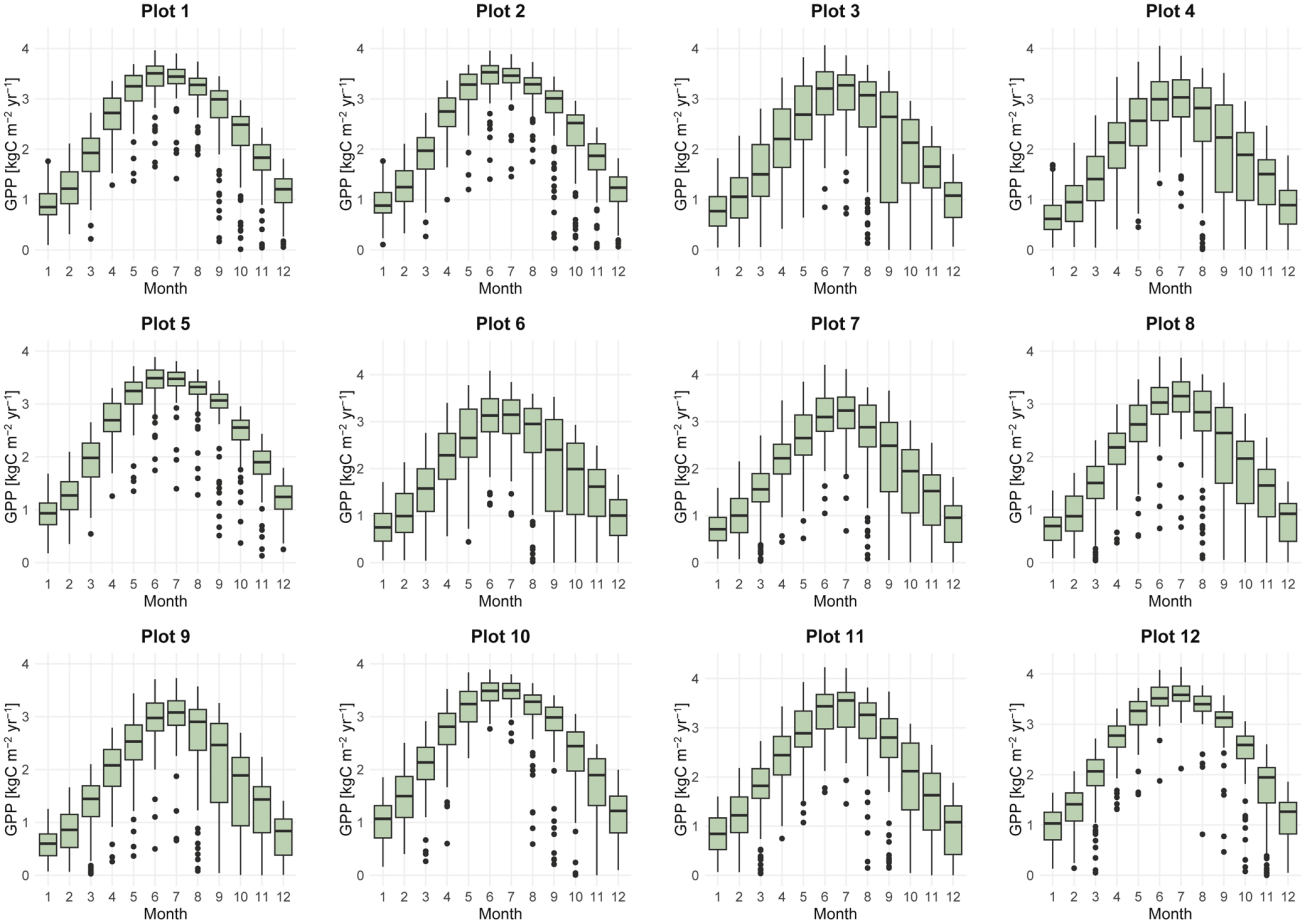
Monthly and yearly variability of simulated gross primary production (kg C m^−2^ year^−1^) during stand development (i.e., 1–97 simulation years) from ED‐2.2 model simulation. In ED‐2.2, leaf physiology is modeled following Farquhar et al. (1980) and Collatz et al. (1991); and Leuning (1995) model for stomatal conductance.

**FIGURE 4:**
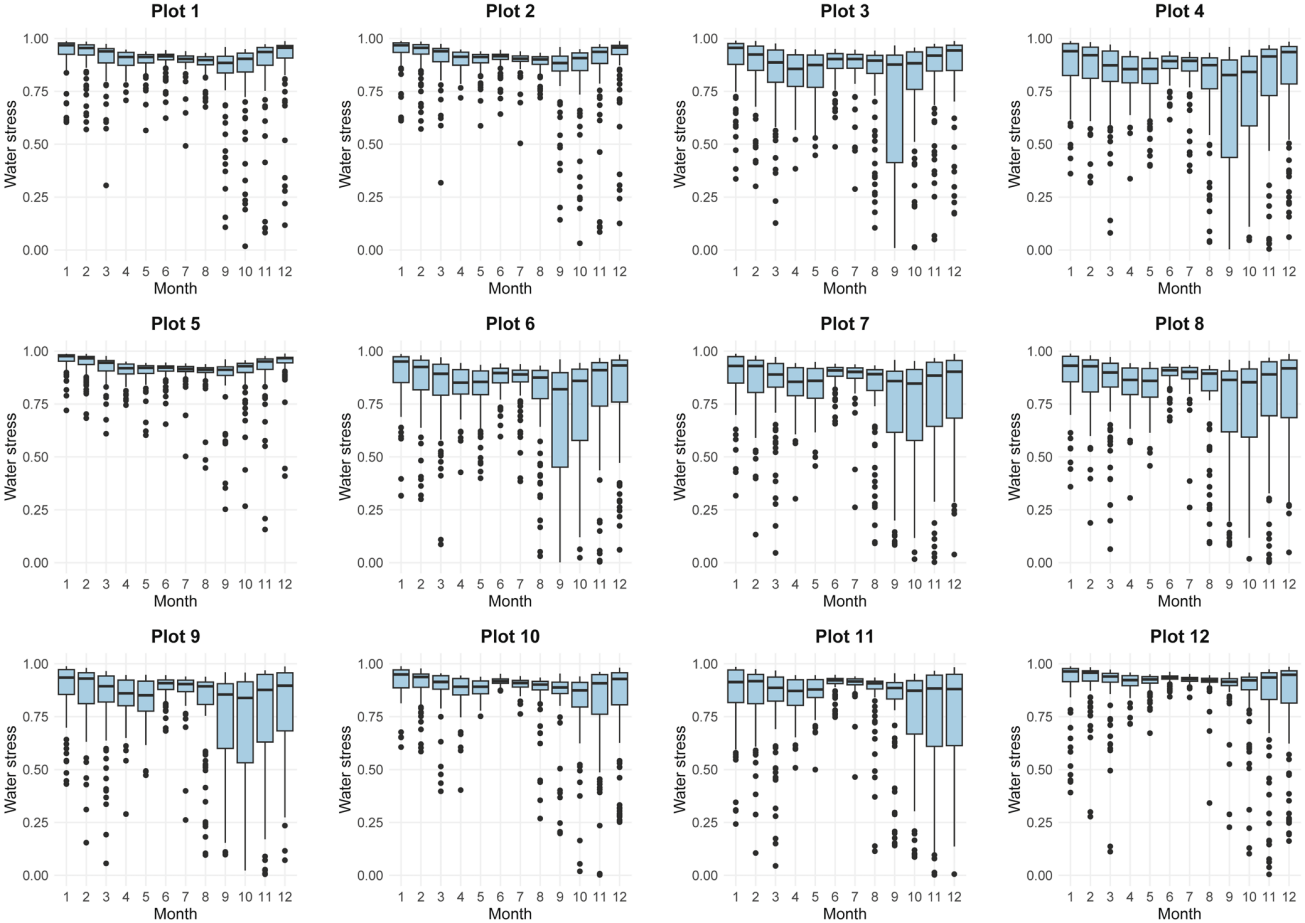
Monthly and yearly variability of simulated water stress factor during stand development (i.e., 1–97 simulation years) from ED‐2.2 model simulation. Because the stomatal conductance model by Leuning (1995) is regulated only by leaf vapor pressure deficit, in order to account for soil moisture limitation of photosynthesis, water stress factor (i.e., “wilting point”, weighting factor for open (1.00) and closed stomata (0.00)) is defined to reduce productivity as soil available water decreases in ED‐2.2. This factor determines the fraction of open stomata due to soil moisture limitation.

**FIGURE 5:**
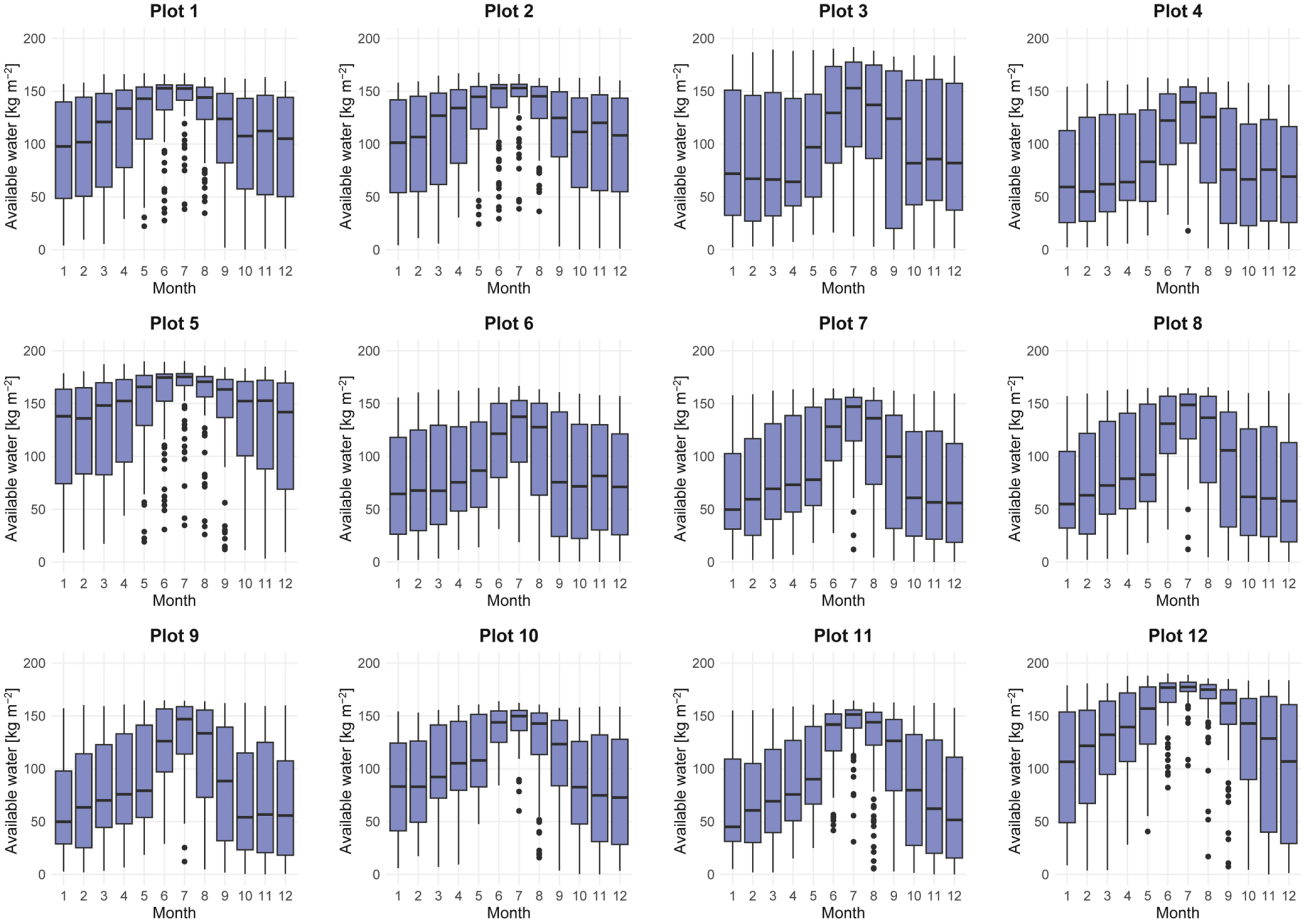
Monthly and yearly variability of simulated available water (kg m^−2^) during stand development (i.e., 1–97 simulation years) from ED‐2.2 model simulation. Available water is defined as the soil moisture above water stress factor (i.e., “wilting point”), scaled by liquid water fraction in ED‐2.2.

Across nearly all plots, air temperature, shortwave radiation, simulated leaf temperature and photosynthetically active radiation (PAR) absorbed by leaves exhibited consistently low interannual variability across all months, with the exception of leaf temperature during winter (Figures S4–S6, S8). Precipitation rates across almost all plots were lower between July and September compared to April to June, with July exhibiting greater interannual variability in precipitation rate than other months (Figure S7). However, according to box plots and median values, the precipitation rate in Group 1 plots was generally higher than that in Group 2 plots (Figure S9). Air temperature and leaf temperature were lower in Group 1 plots than in Group 2 plots (Figure S9). Precipitation rate, available water, water stress factor, and leaf transpiration were higher in Group 1 plots than in Group 2 plots (Figure S9). However, no clear differences were observed in shortwave radiation and photosynthetically active radiation (PAR) absorbed by leaves between Group 1 and Group 2 plots (Figure S9).

Moreover, simulated density‐dependent mortality rates were consistently higher than senescence‐induced mortality (Figure 6). The high mortality rates for density‐dependent were particularly higher during the summer‐autumn transition (especially in September and October) compared to other months (Figure 6). In contrast, the low‐to‐moderate rates for simulated density‐dependent mortality showed no evident interannual and seasonal variability (Figure 6). There were no clear differences in shortwave radiation and PAR absorbed by leaves between the two plot groups (Figures S5 and S8), whereas precipitation and water‐related intermediate variables (available water, water‐stress factor) differ substantially (Figures 4, 5, and Figure S7). Consequently, the higher simulated density‐dependent and combined mortality in late summer‐autumn is primarily drought‐driven (precipitation stress) rather than light competition‐driven.

**FIGURE 6:**
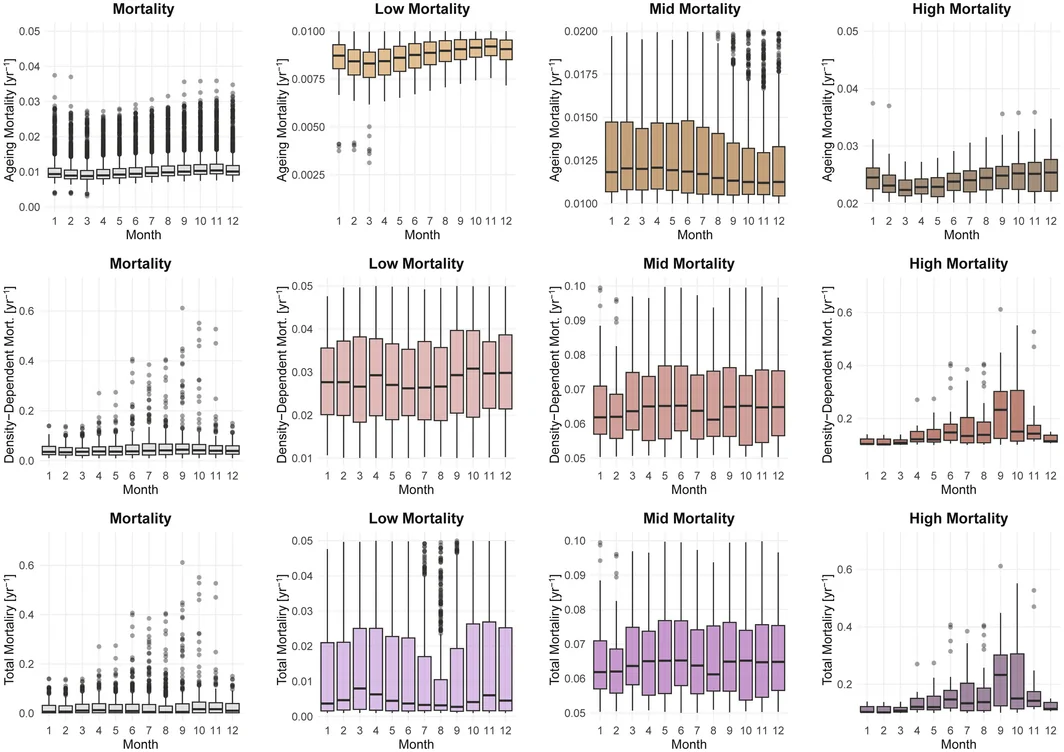
Monthly and yearly variability of simulated density‐independent (aging), density‐dependent (negative carbon balance), and combined tree mortality rates (year^−1^) during stand development (i.e., 1–97 simulation years) from ED‐2.2 model simulation. Density‐independent mortality rate was attributed to the component of cohort aging, was categorized as low (0.00–0.01), mid (0.01–0.02), and high (> 0.02). Density‐dependent mortality rate described the limitations of carbon uptake due to resource competition for light and water, was classified as low (0.01–0.05), mid (0.05–0.10), and high (> 0.10). Combined tree mortality was the sum of density‐independent and density‐dependent mortality, was categorized as low (0.00–0.05), mid (0.05–0.10), and high (> 0.10). The combined tree mortality rate was almost entirely contributed by density‐dependent mortality.

When we generalized the results by pooling all plots together, we found that the overall pattern fell between that of the Group 1 plots and the Group 2 plots (Figure S10). For example, AGB was relatively high during the early period (similar to Group 1 plots) but declined markedly in the later period (similar to Group 2 plots). The interannual variability of GPP, water stress factor, and available water in late summer‐autumn was generally larger than that in Group 1 plots but smaller than that in Group 2 plots.

## 4 Discussion

### 4.1 Stability of Forest Productivity Declines During Development

The simulated stand development trajectory revealed a general decline in stability of forest productivity over time (Tables 1, 2, 3 and Tables S6–S10), implying that this pattern was driven by concurrent shifts in the composition and structure of subtropical coniferous forests (Figures 1 and 2). The species composition of stands typically transitioned from a mixture of coniferous and broadleaved trees at early stages to dominated by either light‐demanding conifers or shade‐tolerant broadleaves in later stages (Figure 1). In the later stages, the AGB of the light‐demanding broadleaved trees (PFT2) was nearly zero. This shift may result in a markedly reduction in PFT richness and evenness.

PFT richness, which was in analogy to species richness, representing the number of co‐occurring PFTs in the community (Plekhanova et al. 2021; Schmid et al. 2021). Similarly, PFT evenness can be understood as reflecting the uneven aboveground biomass distribution between light‐demanding conifer (PFT7) and shade‐tolerant broadleaf (PFT4) within the forest ecosystem. Conifers typically exhibit lower drought tolerance than broadleaved species (Zhang et al. 2023). Consequently, when ecosystem AGB was predominantly contributed by PFT7, seasonal drought during summer and autumn likely triggered tree mortality, leading to AGB decline and reduced temporal stability of annual productivity. Similarly, the stand structure showed a clear development trajectory: a relatively balanced diameter distribution in early stages often became increasingly dominated by either large or small‐to‐middle trees as stands matured (Figure 2), leading to a pronounced simplification of structural complexity. When AGB is mainly contributed by large light‐demanding conifers, structural simplification means that when these large trees die (likely associated with aging and seasonal drought), AGB will decline sharply, negatively affecting temporal stability of productivity.

Forest productivity and stability were enhanced by tree diversity and stand structure through the complementarity effect of niche partitioning, as demonstrated in multiple BEF studies (Ding et al. 2024; LaRue et al. 2023; Liu, Feng, et al. 2024; Ray et al. 2023; Zhai et al. 2024). The findings of our study were similar to previous research. The positive diversity‐stability relationships in forests were consistently demonstrated across a range of forest ecosystems. This pattern was identified in model virtual experiments for Central European forests (Morin et al. 2014), observed across Canadian boreal and temperate forests (Ding et al. 2024), and confirmed by a tropical tree diversity experiment in Panama (Schnabel et al. 2019). Furthermore, Shan et al. (2025) illustrated this relationship in eastern US forests, reporting that a decline in productivity stability with forest age was linked to simplification of community composition, which in our study was represented in a simplified way by PFT richness. Numerous studies across diverse forest ecosystems reported a positive effect of structural attributes on forest stability. At the regional scale, Ouyang et al. (2020) found that structural complexity enhanced stability in subtropical forests of China, where more complex diameter structures improved light capture and use efficiency. Similarly, Roces‐Díaz et al. (2025) reported a positive effect of stand structure, particularly maximum DBH, on stability in Mediterranean‐temperate mixed forests, while Dolezal et al. (2020) documented that higher stand density promoted stability in a mixed forest in Hokkaido, Japan. Liu, Feng, et al. (2024) provided global‐scale evidence using airborne and spaceborne lidar data, showing that higher canopy structural complexity was associated with stronger productivity stability in global forests. Collectively, these studies underscored the crucial role of structural complexity and complexity in maintaining forest ecosystem stability.

Our study expands the understanding of temporal changes in forest stability, finding that the stability in subtropical coniferous forests is driven by long‐term dynamics in forest composition and structure.

### 4.2 Precipitation Plays a Key Role in Shaping Stability

For plots exhibiting simulated low productivity stability throughout stand development (e.g., Plots 7–9, and 11), the pattern were likely associated with seasonal precipitation stress between summer and autumn in subtropical China rather than air temperature or atmospheric radiation (Figures 3, 4, 5, Figures S4–S9). Seasonal precipitation stress may be linked to a cascade of effects, ranging from reduced stomatal conductance (i.e., “wilting point”, weighting factor for open and closed stomata, 0–1) and available water to diminished leaf photosynthesis and gross primary production (Figures 3, 4, 5) (Longo et al. 2019; Medvigy et al. 2009; Moorcroft et al. 2001). This cascade ultimately may increase density‐dependent tree mortality, potentially altering forest composition and structure, and contributing to reduced productivity stability (Figures 1, 2, 6, Tables 1, 2, 3, S6–S10) (Bugmann et al. 2019; Hartmann et al. 2018). From July, there was a sharp decline in precipitation rate for three consecutive months (July–September, Figure S7), but severe moisture limitation likely associated with GPP reduction did not occur until September (Figure 3), which may reflect a “lag effect” of drought on tree growth (Huang et al. 2021). Although all plots experienced a sharp drop in precipitation rates for three consecutive months starting in July (Figure S7), not all plots necessarily developed severe moisture deficits likely associated with GPP reduction (Figure 3). This requires comprehensive assessment combined with annual precipitation rates (Figure S9). In addition, air temperature might have also intensified drought‐induced water stress (Figure S9). Warming‐induced drought has been found to limit tree growth in subtropical forests of China from tree‐rings evidence (Jing et al. 2022; Zhang et al. 2023).

A growing body of evidence underscored the critical role of water availability in regulating forest ecosystem stability. Roces‐Díaz et al. (2025) identified water availability as the strongest positive driver of ecosystem stability in Mediterranean‐temperate forests. In these systems, where moisture is typically limited (as indicated by a low precipitation‐to‐potential‐evapotranspiration ratio, P/PET), P/PET was the most significant predictor of stability. This finding underscored the fundamental role of water availability in maintaining forest ecosystem stability (Qiao et al. 2023a, 2023b). Ouyang et al. (2020) demonstrated that precipitation variability indirectly influenced the stability of productivity by mediating changes in both tree species richness and structural complexity in subtropical China. Huang and Xia (2019) revealed higher productivity stability in global forests of humid ecoregions compared to arid ecoregions using a remotely sensed time series data. Yan et al. (2023) demonstrated that drought stress can reduce forest vitality, potentially leading to tree mortality, and ultimately compromise ecosystem stability in subtropical China using the process‐based modeling evidences. Liu et al. (2022) demonstrated that tree species diversity enhances drought resistance in subtropical dry forests, thereby maintaining their stability by integrating forest plot and satellite remotely sensed data. Ding et al. (2024) showed a positive correlation between productivity stability and an aridity index (defined as the ratio of precipitation to evapotranspiration) in Canadian forests. Collectively, these studies highlight water availability as a fundamental regulator of forest stability while also emphasizing the important buffering role of PFT diversity in mitigating drought effects.

### 4.3 Implications and Limitations

A key insight from existing literatures may be the superior drought resistance of evergreen broadleaf forests (EBF) over evergreen coniferous forests (ECF) in subtropical China. This pattern was observed both globally using enhanced vegetation index (Huang and Xia 2019) and regionally using carbon storage (Yan et al. 2023), and was explained by the higher photosynthetic efficiency that EBF maintained during drought. This physiological advantage allowed EBF to better adapt to subtropical droughts, resulting in higher ecosystem stability. Liu et al. (2022) found that species diversity enhanced forest resistance to drought globally, validating the management strategy of converting monocultures into mixtures. This approach holds significant potential for improving drought resilience, particularly in water‐limited systems like subtropical dry forests. Consequently, establishing mixed‐species plantations represented a crucial management strategy for bolstering forest stability under climate change, especially in subtropical coniferous forests.

While parameter sensitivity analysis in this study supported the robustness of our key findings, future work should incorporate trait plasticity, allowing key physiological parameters (e.g., SLA and V_cmax_) to dynamically respond to light availability (e.g., SLA and V_cmax_) (Keenan and Niinemets 2016; Lloyd et al. 2010; Russo and Kitajima 2016). In addition, although simulated AGB was evaluated against independent field observations, we did not quantitatively validate monthly fluxes variables (e.g., GPP, available water, water‐stress) because corresponding site‐level monthly observations were not available. Accordingly, our interpretation that the decline in productivity stability is associated with seasonal precipitation stress between summer and autumn should be regarded as a model‐based hypothesis supported by monthly variables (MERRA‐2 reanalysis product and ED‐2.2 simulated variables), rather than a fully observation‐constrained attribution. Once monthly observations become available, future work should directly test these proposed monthly mechanisms by integrating eddy covariance flux measurements (e.g., GPP), soil‐moisture monitoring, and transpiration‐related observations at representative subtropical coniferous sites, enabling a more comprehensive quantitative evaluation of the model performance.

In ED‐2.2 model, density‐dependent mortality is parameterized using a semi‐empirical logistic function based on carbon balance, reflecting carbon uptake limitations due to competition for light and water (Longo et al. 2019). However, the current implementation does not account for nutrient limitation (e.g., nitrogen, phosphorus), which may influence tree growth and mortality in subtropical forests. Mortality formulation is a key driver of long‐term forest dynamics for most models (Bugmann et al. 2019). When the biomass lost to tree mortality exceeds the biomass gained from tree growth, annual productivity can be negative, which might further affect long‐term stability outcomes.

Despite belonging to the same PFT, differences still exist between Chinese fir ( *Cunninghamia lanceolata* ) and Masson pine ( *Pinus massoniana* ). Based on field measurements, future efforts could focus on collecting or measuring species‐specific parameter data for Chinese fir and Masson pine separately, and treating them as two distinct PFTs in the modeling studies.

We used sequentially recycled the historical MERRA‐2 reanalysis data to force ED‐2.2 model, however, this simplification may not capture the intensification and increasing complexity of projected drought regimes. Emerging drought patterns (e.g., flash droughts, multi‐year events, and warming‐enhanced droughts) were expected to become more prevalent (Chen et al. 2025; Gu et al. 2025; Yuan et al. 2023). Consequently, these complex stressors could drive forest AGB along more complicated and unpredictable temporal trajectories than those simulated here. This study has examined the stability of subtropical coniferous forests under a baseline scenario without anthropogenic management by virtual experiment simulations. This simulation baseline can expand our understanding of combined effects from anthropogenic management and climate extremes on forest stability. A valuable next step would be to compare these findings with scenarios incorporating anthropogenic management. For instance, future studies could investigate whether the conversion to subtropical mixed broadleaf‐coniferous forests leads to improved productivity stability under emerging drought types. This approach was supported by recent virtual experiments demonstrating that switching coniferous forests to broadleaved forests can effectively mitigate summer hot extremes in European forests from modeling evidences (Yao et al. 2025).

## 5 Conclusion

This study employed a process‐based Ecosystem Demography model (ED‐2.2) to simulate the long‐term stability of aboveground biomass and productivity without anthropogenic management in subtropical coniferous forests of China. The simulated stand development trajectory indicated a general decline in forest productivity stability over time in the absence of management, suggesting that this pattern is probably driven by concurrent shifts in the composition and structure of subtropical coniferous forests. In plots exhibiting low stability of forest productivity throughout stand development, this was likely associated with seasonal precipitation stress occurring between summer and autumn. Our findings enhance the understanding of temporal dynamics in forest stability, demonstrating that the stability of subtropical coniferous forests is modulated by long‐term changes in forest composition and structure, primarily through the constraint of seasonal precipitation.

## Author Contributions

**Jiajia Wang:** data curation (lead), formal analysis (lead), investigation (equal), methodology (equal), software (equal), validation (lead), visualization (lead), writing – original draft (equal), writing – review and editing (equal). **Yu Zhu:** conceptualization (lead), funding acquisition (equal), investigation (equal), project administration (equal), supervision (lead), writing – review and editing (lead). **Shuguang Liu:** formal analysis (equal), funding acquisition (equal), resources (equal), supervision (equal), writing – review and editing (equal). **Quanlin Li:** formal analysis (equal), software (equal), writing – review and editing (equal). **Zhao Wang:** formal analysis (equal), software (equal), writing – review and editing (equal). **Meifang Zhao:** formal analysis (equal), supervision (equal), writing – review and editing (equal). **Liangjun Zhu:** formal analysis (equal), supervision (equal), writing – review and editing (equal).

## Funding

This research was funded by the National Natural Science Foundation of China (31901241, U20A2089, 41971152), the Research Foundation of Education Bureau of Hunan Province, China (21B0277), and the Start‐up Scientific Research Foundation for the Introduction of Talents in Central South University of Forestry and Technology (2018YJ010).

## Conflicts of Interest

The authors declare no conflicts of interest.

## Supporting information


**Table S1:** Summary of forest inventory plots.
**Table S2:** Six theoretical growth equations for DBH‐height allometry.
**Table S3:** Performance of original vs. modified ED‐2.2 models in simulating aboveground biomass which height‐DBH allometric equations were modified by six growth equations. RMSE, root mean square error. MAE, mean absolute error. Φ, weighted least squares method.
**Table S4:** Performance of 50 ensemble runs sampling parameters from the posterior distribution vs. 10 ensemble runs sampling parameters from the prior distribution in simulating aboveground biomass. RMSE, root mean square error. MAE, mean absolute error. Φ, weighted least squares method. Parameters: SLA, specific leaf area. V_cmax15_, maximum Rubisco carboxylation rate at 15°C for subtropical light‐demanding conifers PFT in ED‐2.2.
**Table S5:** Eco‐physiological, life‐history, and allometric parameters for the plant functional types. Optimized and default parameters are in and out parentheses, respectively. SLA, specific leaf area. V_cmax15_, maximum Rubisco carboxylation rate at 15°C. WD, wood density. M_DI_, density‐independent mortality. b1Ht and b2Ht, DBH‐height allometric coefficients. Parameter data were collected through literatures search (Chave et al. 2009; Dong 2015; Dong et al. 2015; Kattge et al. 2020; LeBauer et al. 2018; Li and Xiang 2011; Li et al. 2007; Li 2016; Li 2019; Li et al. 2018; Paine et al. 2015; Prentice et al. 2011; Wang et al. 2022; Wang et al. 2018; Wang 2010; Wu et al. 2021; Xie et al. 2022; Xu, Wang, Prentice, Harrison, Wang, and Sun 2021; Xu, Wang, Prentice, Harrison, and Wright 2021; Ye 2019; Zhou 2013; Zhu et al. 2022).
**Table S6:** Temporal stability of simulated aboveground biomass and forest productivity during stand development (i.e., 1–20 simulation years).
**Table S7:** Temporal stability of simulated aboveground biomass and forest productivity during stand development (i.e., 21–40 simulation years).
**Table S8:** Temporal stability of simulated aboveground biomass and forest productivity during stand development (i.e., 41–60 simulation years).
**Table S9:** Temporal stability of simulated aboveground biomass and forest productivity during stand development (i.e., 61–80 simulation years).
**Table S10:** Temporal stability of simulated aboveground biomass and forest productivity during stand development (i.e., 81–97 simulation years).
**Table S11:** Sensitivity of simulated aboveground biomass to specific leaf area (SLA) and maximum Rubisco carboxylation rate at 15°C (V_cmax15_) for subtropical coniferous plant functional type (PFT7).
**Figure S1:** Sensitivity of simulated aboveground biomass to variations in the specific leaf area (SLA) parameter for subtropical coniferous plant functional type (PFT7).
**Figure S2:** Sensitivity of simulated aboveground biomass to variations in the maximum Rubisco carboxylation rate at 15°C (V_cmax15_) parameter for subtropical coniferous plant functional type (PFT7).
**Figure S3:** Monthly and yearly variability of simulated leaf transpiration (kg m^−2^ day^−1^) during forest development (2004–2100) from ED‐2.2 model simulation.
**Figure S4:** Monthly and yearly variability of simulated leaf temperature (K) during forest development (2004–2100) from ED‐2.2 model simulation.
**Figure S5:** Monthly and yearly variability of simulated PAR absorbed by leaves (W m^−2^) during forest development (2004–2100) from ED‐2.2 model simulation.
**Figure S6:** Monthly and yearly variability of air temperature (K) during forest development (2004–2100) from MERRA‐2 reanalysis data.
**Figure S7:** Monthly and yearly variability of precipitation rate (kg m^−2^ day^−1^) during forest development (2004–2100) from MERRA‐2 reanalysis data.
**Figure S8:** Monthly and yearly variability of shortwave radiation (W m^−2^) during forest development (2004–2100) from MERRA‐2 reanalysis data.
**Figure S9:** Variability of meteorological (i.e., air temperature, precipitation rate, and shortwave radiation) and simulated intermediate (i.e., water stress, leaf temperature, available water, PAR absorbed by leaves, and leaf transpiration) variables among plots during forest development (2004–2100) from MERRA‐2 reanalysis data and ED‐2.2 model simulation, respectively.
**Figure S10:** Long‐term trajectory of simulated (A) forest composition, (B) structure, and aboveground biomass (kg C m^−2^) and variability of simulated (C) gross primary production (kg C m^−2^ year^−1^), (D) water stress factor, (E) available water (kg m^−2^), (F) leaf transpiration (kg m^−2^ day^−1^), (G) leaf temperature (K), (H) PAR absorbed by leaves (W m^−2^), (I) air temperature (K), (J) precipitation rate (kg m^−2^ day^−1^), and (K) shortwave radiation (W m^−2^) for all plots during stand development (i.e., 1–97 simulation years) from ED‐2.2 model simulation. Gray indicates total trees; purple indicates light‐demanding coniferous trees (PFT7); dark green indicates shade‐tolerant evergreen broadleaved trees (PFT4); light green indicates light‐demanding evergreen broadleaved trees (PFT2). Gray indicates total trees; blue indicates large‐sized trees (DBH > 30 cm); dark red indicates medium‐sized trees (10 cm < DBH ≤ 30 cm); light red indicates small‐sized trees (DBH ≤ 10 cm).
**Table S12:** Absolute temporal stability of simulated aboveground biomass and forest productivity during stand development (i.e., 1–97 simulation years).
**Table S13:** Absolute temporal stability of simulated aboveground biomass and forest productivity during early development period (i.e., 1–50 simulation years).
**Table S14:** Absolute temporal stability of simulated aboveground biomass and forest productivity during late development period (i.e., 51–97 simulation years).
**Table S15:** Detrended temporal stability of simulated aboveground biomass and forest productivity during stand development (i.e., 1–97 simulation years).
**Table S16:** Detrended temporal stability of simulated aboveground biomass and forest productivity during early development period (i.e., 1–50 simulation years).
**Table S17:** Detrended temporal stability of simulated aboveground biomass and forest productivity during late development period (i.e., 51–97 simulation years).

## Data Availability

The data that support the findings of this study are available from the Dryad Digital Repository: https://doi.org/10.5061/dryad.gb5mkkx34.
