# Supplementary material for: Long‐Term Stability of Aboveground Biomass and Productivity Without Anthropogenic Management in Subtropical Coniferous Forests: Insights From the Ecosystem Demography Model
Source: Ecol Evol. 2026 Nov 13;16(11):e74660. doi: 10.1002/ece3.74660 (PMC13768456; doi:10.1002/ece3.74660)
Supplement: Supplementary file 1 [file ECE3-16-e74660-s001.docx]

## *Supporting information of*

## “Long-term stability of aboveground biomass and productivity without anthropogenic management in subtropical coniferous forests: Insights from the Ecosystem Demography model”

**Jiajia Wang ^1,2^, Yu Zhu ^1,2,3^**^*^**, Shuguang Liu ^4^, Quanlin Li ^1,2^, Zhao Wang ^1,2^, Meifang Zhao ^1,2^, Liangjun Zhu ^5^.**

^1^ School of Ecology and Environment, Central South University of Forestry and Technology, Changsha 410004, China;

^2^ National Engineering Laboratory for Applied Technology of Forestry & Ecology in South China, Central South University of Forestry and Technology, Changsha 410004, China;

^3^ Technology Innovation Center for Ecological Conservation and Restoration in Dongting Lake Basin, Ministry of Natural Resources, Changsha 410004, China;

^4^ School of Ecology, Hainan University, Haikou 570228, China;

^5^ School of Ecology, Northeast Forestry University, Harbin 150040, China.

*Corresponding author: Yu Zhu ([koxinga_zhu@csuft.edu.cn](mailto:koxinga_zhu@csuft.edu.cn)).

## S1. Forest inventory plot

**Table S1. Summary of forest inventory plots.**

| **Plot** | **Dominant Species** | **Altitude (m)** | **Stand Age (a)** | **Mean DBH (cm)** | **Mean Height (m)** |
| --- | --- | --- | --- | --- | --- |
| 1 | *C. lanceolata* | 250 | 9 | 7.8 | 6.1 |
| 2 | *P. massoniana* | 340 | 13 | 9.5 | 6.8 |
| 3 | *P. massoniana* | 240 | 18 | 9.7 | 8.6 |
| 4 | *P. massoniana* | 350 | 30 | 26.8 | 12.5 |
| 5 | *C. lanceolata* | 380 | 10 | 10.4 | 8.0 |
| 6 | *C. lanceolata* | 270 | 7 | 6.5 | 7.2 |
| 7 | *C. lanceolata* | 480 | 18 | 15.0 | 11.1 |
| 8 | *C. lanceolata* | 580 | 10 | 10.7 | 7.4 |
| 9 | *C. lanceolata* | 450 | 8 | 9.1 | 8.3 |
| 10 | *P. massoniana* | 430 | 28 | 24.8 | 18.2 |
| 11 | *C. lanceolata* | 460 | 19 | 19.9 | 16.4 |
| 12 | *P. massoniana* | 490 | 6 | 7.5 | 5.0 |

## S2. Optimization on DBH-height allometry

In the release version of the ED-2.2 model, the relationship between tree height and diameter at breast height (DBH) for subtropical coniferous species (PFT7) is described solely by a modified Weibull allometric equation. This approach, however, introduces regional variations that compromise the accuracy of biomass predictions. In this study, we selected six widely recognized theoretical growth equations as candidate models for DBH-height allometry: Richards (Richards, 1959), Korf (Kiviste, 1988), Logistic (Verhulst, 1838), Gompertz (Gompertz, 1997), Weibull (Weibull, 1951), and Mitscherlich (Mitscherlich, 1909). We extracted all available measurements of DBH and heights of individual trees for *Cunninghamia lanceolata* and *Pinus massoniana* forests in China from the Tallo tree allometry database (Jucker et al., 2022). The filtered dataset, comprising 216 records, was used to fit the six allometric equations. The resulting fitted models (Table S2) were then individually integrated into the ED-2.2 model. Consequently, seven variants of the ED-2.2 model were employed in this study: the original ED-2.2 (Release version), along with ED-2.2 (Richards), ED-2.2 (Korf), ED-2.2 (Logistic), ED-2.2 (Gompertz), ED-2.2 (Weibull), and ED-2.2 (Mitscherlich). Simulation accuracy was assessed using the mean absolute error (MAE), root mean square error (RMSE), and the performance index Φ based on weighted least squares (Liu et al., 2008). Among them, the ED-2.2 (Korf) model produced the smallest errors (MAE = 0.932; RMSE = 1.099; Φ = 1.679), while the ED-2.2 (Release version) model showed the largest (MAE = 1.046; RMSE = 1.219; Φ = 2.056) (Table S3). Overall, all six modified models outperformed the original release in simulating AGB, with the ED-2.2 (Korf) version exhibiting the best performance (Table S2). All the statistical computing and graphics were implemented in R v.4.1.1 (R_Core_Team, 2021).

**Table S2.** **Six theoretical growth equations for DBH-height allometry.**

| **Number** | **Equation Type** | **Equation** |
| --- | --- | --- |
| 1 | Logistic | $HGT=\frac{25.532}{1+4.098\cdot e^{-0.068\cdot DBH}}$ |
| 2 | Korf | $HGT=10160\cdot(e^{\frac{-9.092}{{DBH}^{0.099}}}）$ |
| 3 | Weibull | $HGT=491.2\cdot(1-e^{-0.004\cdot{DBH}^{0.604}})$ |
| 4 | Mitscherlich | $HGT=35.654\cdot(1-0.921\cdot e^{-0.018\cdot DBH})$ |
| 5 | Gompertz | $HGT=27.859\cdot(e^{-1.881\cdot e^{-0.043\cdot DBH}})$ |
| 6 | Richards | $HGT=344.1\text{∙}{(1-e^{-0.0002\cdot DBH})}^{0.597}$ |

**Table S3. Performance of original vs. modified ED-2.2 models in simulating aboveground biomass which height-DBH allometric equations were modified by six growth equations.** RMSE, root mean square error. MAE, mean absolute error. Φ, weighted least squares method.

| **Equation Type** | **MAE** | **RMSE** | $\boldsymbol{\Phi}$ |
| --- | --- | --- | --- |
| Release Version | 1.046 | 1.219 | 2.056 |
| Korf | 0.932 | 1.099 | 1.679 |
| Weibull | 1.021 | 1.193 | 1.969 |
| Richards | 1.022 | 1.194 | 1.972 |
| Logistic | 1.044 | 1.216 | 2.041 |
| Gompertz | 1.043 | 1.215 | 2.039 |
| Mitscherlich | 1.041 | 1.213 | 2.033 |

## S3. Model parameterization and validation

Parameter optimization for plant functional types (PFTs) has traditionally involved iterative sampling from prior parameter distributions. Recently, however, Bayesian meta-analysis has been employed to derive a more constrained posterior distribution, from which parameters are subsequently sampled for optimization (Feng et al., 2018; Shiklomanov, Dietze, Fer, Viskari, & Serbin, 2021). This study aims to compare the effectiveness of these two approaches in obtaining optimal parameter sets. We collected species level parameter data for subtropical light-demanding conifers PFT (Chave et al., 2009; Dong, Zhang, Zhang, & Zhang, 2015; Kattge et al., 2020; D. LeBauer et al., 2018; Yue Li, Zhou, Wang, Chi, & Chen, 2018; Paine et al., 2015; Prentice et al., 2011; H. Wang et al., 2022; Han Wang et al., 2018; Wu et al., 2021), including specific leaf area (SLA; in m^2^ kg⁻^1^) and the maximum carboxylation rate of Rubisco (V_cmax_; in μmol m⁻^2^ s⁻^1^). These data were incorporated into a local instance of the BETYdb database for Bayesian meta-analysis using the PEcAn toolbox (Dietze, Lebauer, & Kooper, 2013; D. LeBauer et al., 2018; D. S. LeBauer, Wang, Richter, Davidson, & Dietze, 2013). We first sampled parameters 50 times from the converged posterior distribution. Then, an additional 10 parameters were sampled from the prior distribution (excluding values already drawn from the posterior). Finally, the performance of the two parameter-sampling approaches was compared to determine which produced superior results.

The 2004 inventory data are single‑time measurements, which was sufficient to constrain the initial forest structure and composition but did not constitute a repeated-measurement time series for evaluating multi-decadal AGB trajectories at the same plots. To evaluate model performance, we used a dataset derived from the same forest inventory data in Hunan Province, China (C. Wang et al., 2024), which provided age‑AGB relationships for Chinese fir forests across a broad age range (C. Wang et al., 2024). Following a chronosequence approach (space‑for‑time substitution) (Zhao et al., 2013), we validated the simulated AGB as a function of stand age against the inventory-based stand age‑AGB relationship. Model performance was evaluated against observed aboveground biomass data from subtropical coniferous forests of different age classes in Hunan Province, China (C. Wang et al., 2024). We obtained posterior distributions of specific leaf area (SLA) and the maximum rate of Rubisco carboxylase activity at 25 °C (V_cmax25_) for light-demanding conifers (PFT7) through Bayesian meta-analysis. Fifty parameter sets were converted into ED-2.2 model-compatible units (SLA in m^2^ kg⁻^1^ C; V_cmax15_ in μmol m⁻^2^ s⁻^1^). Using the optimized ED-2.2 (Korf) model, 50 ensemble runs were conducted per plot. The resulting 12,000 simulated aboveground biomass values were aligned with theoretical stand ages and aggregated for comparison with observed data. Among the 50 simulations sampling parameters from the posterior distribution, the optimal parameter values were SLA = 17.52 m^2^ kg⁻^1^ C and V_cmax15_ = 20.94 μmol m⁻^2^ s⁻^1^. An additional 10 ensemble runs were performed using parameters sampled from the prior distribution. The corresponding optimal parameter set was SLA = 10.09 m^2^ kg⁻^1^ C and V_cmax15_ = 19.07 μmol m⁻^2^ s⁻^1^ (Table S5). Parameters sampled from the prior distribution produced lower MAE, RMSE, and Φ values (MAE=0.543, RMSE=0.432, and Φ=0.384) than from the posterior distribution (MAE=0.791, RMSE=0.600, and Φ=0.723), indicating improved simulation accuracy (Table S4). The PFT-dependent parameters (e.g., SLA, specific leaf area; V_cmax_, maximum Rubisco carboxylation rate; WD, wood density; b1Ht and b2Ht, DBH-height allometric coefficients) were obtained from the literatures (Tables S5) (Chave et al., 2009; Dong, 2015; Dong et al., 2015; Kattge et al., 2020; D. LeBauer et al., 2018; K. Li & Xiang, 2011; X.-R. Li, Liu, Cai, & Ma, 2007; Yiyong Li, 2016; Yue Li, 2019; Yue Li et al., 2018; Paine et al., 2015; Prentice et al., 2011; H. Wang et al., 2022; Han Wang et al., 2018; Y. Wang, 2010; Wu et al., 2021; Xie et al., 2022; Xu, Wang, Prentice, Harrison, Wang, et al., 2021; Xu, Wang, Prentice, Harrison, & Wright, 2021; Ye, 2019; Zhou, 2013; Zhu et al., 2022).

**Table S4. Performance of 50 ensemble runs sampling parameters from the posterior distribution vs. 10 ensemble runs sampling parameters from the prior distribution in simulating aboveground biomass.** RMSE, root mean square error. MAE, mean absolute error. Φ, weighted least squares method. Parameters: SLA, specific leaf area. V_cmax15_, maximum Rubisco carboxylation rate at 15°C for subtropical light-demanding conifers PFT in ED-2.2.

| **Distribution** | **MAE** | **RMSE** | $\boldsymbol{\Phi}$ |
| --- | --- | --- | --- |
| Posterior | 0.791 | 0.600 | 0.723 |
| Prior | 0.543 | 0.432 | 0.384 |

**Table S5. Eco-physiological, life-history, and allometric parameters for the plant functional types.** Optimized and default parameters are in and out parentheses, respectively. SLA, specific leaf area. V_cmax15_, maximum Rubisco carboxylation rate at 15°C. WD, wood density. M_DI_, density-independent mortality. b1Ht and b2Ht, DBH-height allometric coefficients. Parameter data were collected through literatures search (Chave et al., 2009; Dong, 2015; Dong et al., 2015; Kattge et al., 2020; D. LeBauer et al., 2018; K. Li & Xiang, 2011; X.-R. Li et al., 2007; Yiyong Li, 2016; Yue Li, 2019; Yue Li et al., 2018; Paine et al., 2015; Prentice et al., 2011; H. Wang et al., 2022; Han Wang et al., 2018; Y. Wang, 2010; Wu et al., 2021; Xie et al., 2022; Xu, Wang, Prentice, Harrison, Wang, et al., 2021; Xu, Wang, Prentice, Harrison, & Wright, 2021; Ye, 2019; Zhou, 2013; Zhu et al., 2022).

| **Property** | **Light-demanding coniferous species**  **(PFT7)** | **Light-demanding evergreen broadleaved species**  **(PFT2)** | **Shade-tolerant evergreen broadleaved species**  **(PFT4)** |
| --- | --- | --- | --- |
| SLA (m^2^ kg⁻^1^ C) | 9.00 **(10.09) *** | 16.0 (24.5) | 9.7 (36.6) |
| V_cmax15_ (μmol m⁻^2^ s⁻^1^) | 11.35 **(19.07) *** | 18.75 | 6.25 |
| WD (g cm^–3^) | — | 0.53 (0.49) | 0.90 (0.64) |
| M_DI_ (yr^–1^) | — | 0.062 (0.068) | 0 (0.043) |
| b1Ht (m) | — | 0.035 (0.026) | 0.035 (0.026) |
| b2Ht (cm^–1^) | — | 0.694 (0.715) | 0.694 (0.717) |

* SLA and V_cmax15_ were calibrated through iterative optimization.

## S4. Temporal stability and sensitivity analysis

**Table S6. Temporal stability of simulated aboveground biomass and forest productivity during stand development (i.e., 1~20 simulation years).**

| **Plot** | | **Aboveground Biomass** | | | **Forest Productivity** | | |
| --- | --- | --- | --- | --- | --- | --- | --- |
|  |  | **Mean** | **SD** | **Stability** | **Mean** | **SD** | **Stability** |
| 1 | | 3.456 | 0.938 | 3.687 | 0.184 | 0.145 | 1.272 |
| 2 | | 3.286 | 1.014 | 3.241 | 0.193 | 0.141 | 1.374 |
| 3 | | 3.358 | 0.795 | 4.224 | 0.157 | 0.163 | 0.966 |
| 4 | | 3.249 | 0.860 | 3.780 | 0.149 | 0.177 | 0.842 |
| 5 | | 2.919 | 1.159 | 2.518 | 0.197 | 0.090 | 2.180 |
| 6 | | 2.360 | 1.003 | 2.354 | 0.169 | 0.156 | 1.078 |
| 7 | | 2.744 | 0.989 | 2.775 | 0.165 | 0.171 | 0.966 |
| 8 | | 1.711 | 0.657 | 2.604 | 0.113 | 0.179 | 0.630 |
| 9 | | 1.618 | 0.588 | 2.751 | 0.102 | 0.189 | 0.539 |
| 10 | | 4.054 | 0.568 | 7.140 | 0.124 | 0.131 | 0.950 |
| 11 | | 2.864 | 1.019 | 2.810 | 0.180 | 0.163 | 1.103 |
| 12 | | 2.297 | 0.717 | 3.204 | 0.145 | 0.157 | 0.923 |
| Total | Mean | 2.826 | 0.859 | 3.424 | 0.157 | 0.155 | 1.069 |
|  | SD | 0.726 | 0.192 | 1.301 | 0.031 | 0.026 | 0.421 |

**Table S7. Temporal stability of simulated aboveground biomass and forest productivity during stand development (i.e., 21~40 simulation years).**

| **Plot** | | **Aboveground Biomass** | | | **Forest Productivity** | | |
| --- | --- | --- | --- | --- | --- | --- | --- |
|  |  | **Mean** | **SD** | **Stability** | **Mean** | **SD** | **Stability** |
| 1 | | 5.725 | 0.672 | 8.517 | 0.117 | 0.102 | 1.148 |
| 2 | | 5.553 | 0.709 | 7.834 | 0.116 | 0.096 | 1.205 |
| 3 | | 4.165 | 0.361 | 11.527 | –0.017 | 0.274 | –0.063 |
| 4 | | 3.477 | 0.909 | 3.824 | –0.042 | 0.522 | –0.080 |
| 5 | | 6.003 | 0.831 | 7.224 | 0.137 | 0.076 | 1.799 |
| 6 | | 3.615 | 0.605 | 5.978 | 0.015 | 0.361 | 0.040 |
| 7 | | 3.672 | 0.734 | 5.003 | –0.002 | 0.391 | –0.006 |
| 8 | | 2.828 | 0.429 | 6.595 | 0.052 | 0.245 | 0.214 |
| 9 | | 2.431 | 0.360 | 6.761 | 0.038 | 0.201 | 0.187 |
| 10 | | 5.911 | 0.546 | 10.825 | 0.109 | 0.205 | 0.531 |
| 11 | | 4.563 | 0.531 | 8.601 | 0.044 | 0.320 | 0.137 |
| 12 | | 5.197 | 0.901 | 5.766 | 0.162 | 0.113 | 1.438 |
| Total | Mean | 4.428 | 0.632 | 7.371 | 0.061 | 0.242 | 0.546 |
|  | SD | 1.243 | 0.194 | 2.255 | 0.066 | 0.138 | 0.667 |

**Table S8. Temporal stability of simulated aboveground biomass and forest productivity during stand development (i.e., 41~60 simulation years).**

| **Plot** | | **Aboveground Biomass** | | | **Forest Productivity** | | |
| --- | --- | --- | --- | --- | --- | --- | --- |
|  |  | **Mean** | **SD** | **Stability** | **Mean** | **SD** | **Stability** |
| 1 | | 8.065 | 0.549 | 14.701 | 0.110 | 0.077 | 1.424 |
| 2 | | 8.008 | 0.576 | 13.893 | 0.115 | 0.075 | 1.525 |
| 3 | | 4.962 | 0.390 | 12.721 | 0.087 | 0.136 | 0.641 |
| 4 | | 3.615 | 0.320 | 11.308 | 0.008 | 0.173 | 0.048 |
| 5 | | 8.680 | 0.658 | 13.199 | 0.125 | 0.046 | 2.690 |
| 6 | | 4.828 | 0.265 | 18.245 | 0.055 | 0.169 | 0.327 |
| 7 | | 4.233 | 0.256 | 16.560 | 0.040 | 0.156 | 0.254 |
| 8 | | 3.837 | 0.260 | 14.734 | 0.046 | 0.152 | 0.301 |
| 9 | | 3.061 | 0.254 | 12.059 | 0.030 | 0.146 | 0.205 |
| 10 | | 7.623 | 0.300 | 25.397 | 0.069 | 0.134 | 0.517 |
| 11 | | 5.785 | 0.356 | 16.248 | 0.080 | 0.145 | 0.551 |
| 12 | | 7.569 | 0.432 | 17.532 | 0.093 | 0.110 | 0.838 |
| Total | Mean | 5.855 | 0.385 | 15.550 | 0.072 | 0.127 | 0.777 |
|  | SD | 2.021 | 0.140 | 3.784 | 0.037 | 0.041 | 0.759 |

**Table S9. Temporal stability of simulated aboveground biomass and forest productivity during stand development (i.e., 61~80 simulation years).**

| **Plot** | | **Aboveground Biomass** | | | **Forest Productivity** | | |
| --- | --- | --- | --- | --- | --- | --- | --- |
|  |  | **Mean** | **SD** | **Stability** | **Mean** | **SD** | **Stability** |
| 1 | | 9.406 | 0.143 | 65.642 | 0.0153 | 0.102 | 0.151 |
| 2 | | 9.517 | 0.186 | 51.090 | 0.025 | 0.105 | 0.241 |
| 3 | | 4.596 | 1.351 | 3.401 | –0.157 | 0.478 | –0.328 |
| 4 | | 2.758 | 0.995 | 2.770 | –0.106 | 0.372 | –0.286 |
| 5 | | 10.315 | 0.191 | 54.131 | 0.032 | 0.085 | 0.372 |
| 6 | | 4.073 | 1.611 | 2.529 | –0.163 | 0.616 | –0.265 |
| 7 | | 3.306 | 1.609 | 2.055 | –0.166 | 0.505 | –0.328 |
| 8 | | 3.101 | 1.449 | 2.140 | –0.147 | 0.461 | –0.319 |
| 9 | | 2.519 | 0.973 | 2.590 | –0.099 | 0.334 | –0.297 |
| 10 | | 7.420 | 1.149 | 6.458 | –0.117 | 0.385 | –0.305 |
| 11 | | 4.785 | 2.471 | 1.936 | –0.258 | 0.684 | –0.378 |
| 12 | | 8.446 | 0.172 | 49.158 | –0.009 | 0.151 | –0.061 |
| Total | Mean | 5.854 | 1.025 | 20.325 | –0.096 | 0.357 | –0.150 |
|  | SD | 2.951 | 0.738 | 25.928 | 0.092 | 0.206 | 0.260 |

**Table S10. Temporal stability of simulated aboveground biomass and forest productivity during stand development (i.e., 81~97 simulation years).**

| **Plot** | | **Aboveground Biomass** | | | **Forest Productivity** | | |
| --- | --- | --- | --- | --- | --- | --- | --- |
|  |  | **Mean** | **SD** | **Stability** | **Mean** | **SD** | **Stability** |
| 1 | | 9.187 | 0.207 | 44.319 | –0.036 | 0.097 | –0.374 |
| 2 | | 9.428 | 0.201 | 46.812 | –0.034 | 0.094 | –0.368 |
| 3 | | 1.666 | 0.245 | 6.803 | –0.022 | 0.194 | –0.113 |
| 4 | | 1.879 | 0.260 | 7.234 | –0.019 | 0.139 | –0.137 |
| 5 | | 10.278 | 0.158 | 65.208 | –0.028 | 0.110 | –0.252 |
| 6 | | 2.255 | 0.344 | 6.559 | –0.041 | 0.147 | –0.276 |
| 7 | | 1.779 | 0.216 | 8.240 | –0.017 | 0.147 | –0.119 |
| 8 | | 1.787 | 0.199 | 8.990 | –0.014 | 0.146 | –0.099 |
| 9 | | 1.881 | 0.232 | 8.106 | –0.016 | 0.148 | –0.105 |
| 10 | | 6.036 | 0.384 | 15.719 | –0.055 | 0.178 | –0.313 |
| 11 | | 2.123 | 0.251 | 8.472 | –0.009 | 0.174 | –0.054 |
| 12 | | 8.223 | 0.263 | 31.253 | –0.042 | 0.143 | –0.295 |
| Total | Mean | 4.710 | 0.247 | 21.476 | –0.028 | 0.143 | –0.209 |
|  | SD | 3.600 | 0.063 | 20.281 | 0.014 | 0.031 | 0.115 |

**Table S11. Sensitivity of simulated aboveground biomass to specific leaf area (SLA) and maximum Rubisco carboxylation rate at 15°C (V_cmax15_) for subtropical coniferous plant functional type (PFT7).**

| **Parameter** | **Δ Parameter** | **Δ AGB** | **Sensitivity** |
| --- | --- | --- | --- |
| SLA | +20% | 0.223 | 1.115 |
|  | +10% | 0.152 | 1.520 |
|  | 0% (Baseline) | 0.000 | 0.000 |
|  | –10% | –0.105 | 1.050 |
|  | –20% | –0.303 | 1.515 |
| V_cmax_ | +20% | –0.038 | –0.190 |
|  | +10% | 0.068 | 0.680 |
|  | 0% (Baseline) | 0.000 | 0.000 |
|  | –10% | 0.047 | –0.470 |
|  | –20% | –0.052 | 0.260 |

**Figure S1.** **Sensitivity of simulated aboveground biomass to variations in the specific leaf area (SLA) parameter for subtropical coniferous plant functional type (PFT7).**

**Figure S2.** **Sensitivity of simulated aboveground biomass to variations in the maximum Rubisco carboxylation rate at 15°C (V_cmax15_) parameter for subtropical coniferous plant functional type (PFT7).**

## S5. Variability for meteorological and intermediate variables

**Figure S3.** **Monthly and yearly variability of simulated leaf transpiration (kg m^-2^ d^-1^) during forest development (2004~2100) from ED-2.2 model simulation.**

**Figure S4.** **Monthly and yearly variability of simulated leaf temperature (K) during forest development (2004~2100) from ED-2.2 model simulation.**

**Figure S5.** **Monthly and yearly variability of simulated PAR absorbed by leaves (W m^-2^) during forest development (2004~2100) from ED-2.2 model simulation.**

**Figure S6.** **Monthly and yearly variability of air temperature (K) during forest development (2004~2100) from MERRA-2 reanalysis data.**

**Figure S7.** **Monthly and yearly variability of precipitation rate (kg m^-2^ d^-1^) during forest development (2004~2100) from MERRA-2 reanalysis data.**

**Figure S8.** **Monthly and yearly variability of shortwave radiation (W m^-2^) during forest development (2004~2100) from MERRA-2 reanalysis data.**

**Figure S9.** **Variability of meteorological (i.e., air temperature, precipitation rate, and shortwave radiation) and simulated intermediate (i.e., water stress, leaf temperature, available water, PAR absorbed by leaves, and leaf transpiration) variables among plots during forest development (2004~2100) from MERRA-2 reanalysis data and ED-2.2 model simulation, respectively.**

**Figure S10. Long-term trajectory of simulated (A) forest composition, (B) structure, and aboveground biomass (kg C m^-2^) and variability of simulated (C) gross primary production (kg C m^-2^ yr^-1^), (D) water stress factor, (E) available water (kg m^-2^), (F) leaf transpiration (kg m^-2^ d^-1^), (G) leaf temperature (K), (H) PAR absorbed by leaves (W m^-2^), (I) air temperature (K), (J) precipitation rate (kg m^-2^ d^-1^), and (K) shortwave radiation (W m^-2^) for all plots during stand development (i.e., 1~97 simulation years) from ED-2.2 model simulation.** Grey indicates total trees; purple indicates light-demanding coniferous trees (PFT7); dark green indicates shade-tolerant evergreen broadleaved trees (PFT4); light green indicates light-demanding evergreen broadleaved trees (PFT2). Grey indicates total trees; blue indicates large-sized trees (DBH> 30cm); dark red indicates medium-sized trees (10cm <DBH≤ 30cm); light red indicates small-sized trees (DBH≤ 10cm).

**Table S12. Absolute temporal stability of simulated aboveground biomass and forest productivity during stand development (i.e., 1~97 simulation years).**

| **Plot** | | **Aboveground Biomass** | | | **Forest Productivity** | | |
| --- | --- | --- | --- | --- | --- | --- | --- |
|  |  | **Mean** | **SD** | **Stability** | **Mean** | **SD** | **Stability** |
| 1 | | 7.118 | 2.345 | 3.035 | 0.081 | 0.130 | 0.626 |
| 2 | | 7.102 | 2.486 | 2.857 | 0.086 | 0.128 | 0.675 |
| 3 | | 3.827 | 1.358 | 2.817 | 0.003 | 0.308 | 0.011 |
| 4 | | 3.044 | 0.955 | 3.187 | 0.001 | 0.316 | 0.005 |
| 5 | | 7.574 | 2.919 | 2.595 | 0.096 | 0.114 | 0.844 |
| 6 | | 3.483 | 1.336 | 2.606 | 0.011 | 0.350 | 0.032 |
| 7 | | 3.209 | 1.226 | 2.617 | 0.006 | 0.320 | 0.020 |
| 8 | | 2.695 | 1.101 | 2.448 | 0.012 | 0.271 | 0.045 |
| 9 | | 2.328 | 0.751 | 3.101 | 0.014 | 0.219 | 0.063 |
| 10 | | 6.230 | 1.457 | 4.277 | 0.030 | 0.239 | 0.127 |
| 11 | | 4.104 | 1.801 | 2.279 | 0.010 | 0.379 | 0.026 |
| 12 | | 6.306 | 2.407 | 2.620 | 0.074 | 0.153 | 0.483 |
| Total | Mean | 4.752 | 1.679 | 2.870 | 0.035 | 0.244 | 0.246 |
|  | SD | 1.953 | 0.699 | 0.519 | 0.037 | 0.094 | 0.315 |

**Table S13. Absolute temporal stability of simulated aboveground biomass and forest productivity during early development period (i.e., 1~50 simulation years).**

| **Plot** | | **Aboveground Biomass** | | | **Forest Productivity** | | |
| --- | --- | --- | --- | --- | --- | --- | --- |
|  |  | **Mean** | **SD** | **Stability** | **Mean** | **SD** | **Stability** |
| 1 | | 5.193 | 1.728 | 3.005 | 0.142 | 0.119 | 1.198 |
| 2 | | 5.042 | 1.774 | 2.842 | 0.146 | 0.116 | 1.253 |
| 3 | | 3.960 | 0.752 | 5.266 | 0.0724 | 0.218 | 0.332 |
| 4 | | 3.474 | 0.806 | 4.311 | 0.046 | 0.353 | 0.131 |
| 5 | | 5.194 | 2.190 | 2.372 | 0.160 | 0.083 | 1.918 |
| 6 | | 3.368 | 1.167 | 2.886 | 0.087 | 0.258 | 0.336 |
| 7 | | 3.436 | 0.964 | 3.563 | 0.064 | 0.283 | 0.225 |
| 8 | | 2.587 | 0.933 | 2.773 | 0.066 | 0.202 | 0.326 |
| 9 | | 2.248 | 0.711 | 3.163 | 0.053 | 0.185 | 0.285 |
| 10 | | 5.472 | 1.373 | 3.985 | 0.102 | 0.164 | 0.623 |
| 11 | | 4.096 | 1.271 | 3.224 | 0.407 | 0.237 | 0.096 |
| 12 | | 4.444 | 2.034 | 2.185 | 0.139 | 0.133 | 1.039 |
| Total | Mean | 4.043 | 1.309 | 3.298 | 0.124 | 0.196 | 0.647 |
|  | SD | 1.059 | 0.514 | 0.869 | 0.098 | 0.079 | 0.574 |

**Table S14. Absolute temporal stability of simulated aboveground biomass and forest productivity during late development period (i.e., 51~97 simulation years).**

| **Plot** | | **Aboveground Biomass** | | | **Forest Productivity** | | |
| --- | --- | --- | --- | --- | --- | --- | --- |
|  |  | **Mean** | **SD** | **Stability** | **Mean** | **SD** | **Stability** |
| 1 | | 9.148 | 0.398 | 23.012 | 0.015 | 0.109 | 0.136 |
| 2 | | 9.276 | 0.464 | 19.979 | 0.022 | 0.109 | 0.198 |
| 3 | | 3.663 | 1.795 | 2.040 | **–**0.073 | 0.372 | 0.196 |
| 4 | | 2.578 | 0.899 | 2.869 | **–**0.044 | 0.271 | 0.161 |
| 5 | | 10.085 | 0.479 | 21.062 | 0.027 | 0.103 | 0.261 |
| 6 | | 3.579 | 1.504 | 2.380 | **–**0.069 | 0.418 | 0.164 |
| 7 | | 2.947 | 1.427 | 2.069 | **–**0.059 | 0.349 | 0.170 |
| 8 | | 2.789 | 1.260 | 2.214 | **–**0.050 | 0.322 | 0.155 |
| 9 | | 2.399 | 0.793 | 3.024 | **–**0.032 | 0.244 | 0.131 |
| 10 | | 7.006 | 1.088 | 6.441 | **–**0.051 | 0.280 | 0.181 |
| 11 | | 4.075 | 2.250 | 1.812 | **–**0.088 | 0.472 | 0.187 |
| 12 | | 8.262 | 0.318 | 25.958 | 0.003 | 0.144 | 0.022 |
| Total | Mean | 5.484 | 1.056 | 9.405 | **–**0.033 | 0.266 | 0.164 |
|  | SD | 3.009 | 0.611 | 9.844 | 0.040 | 0.127 | 0.056 |

**Table S15. Detrended temporal stability of simulated aboveground biomass and forest productivity during stand development (i.e., 1~97 simulation years).**

| **Plot** | | **Aboveground Biomass** | | | **Forest Productivity** | | |
| --- | --- | --- | --- | --- | --- | --- | --- |
|  |  | **Mean** | **SD(Residual)** | **Stability** | **Mean** | **SD(Residual)** | **Stability** |
| 1 | | 7.118 | 0.804 | 8.856 | 0.081 | 0.101 | 0.802 |
| 2 | | 7.102 | 0.782 | 9.086 | 0.086 | 0.099 | 0.867 |
| 3 | | 3.827 | 1.316 | 2.908 | 0.003 | 0.294 | 0.012 |
| 4 | | 3.044 | 0.819 | 3.718 | 0.001 | 0.310 | 0.005 |
| 5 | | 7.574 | 0.934 | 8.110 | 0.096 | 0.082 | 1.179 |
| 6 | | 3.483 | 1.335 | 2.610 | 0.011 | 0.338 | 0.033 |
| 7 | | 3.209 | 1.187 | 2.703 | 0.006 | 0.310 | 0.020 |
| 8 | | 2.695 | 1.098 | 2.455 | 0.012 | 0.263 | 0.047 |
| 9 | | 2.328 | 0.744 | 3.127 | 0.014 | 0.212 | 0.065 |
| 10 | | 6.230 | 1.205 | 5.170 | 0.030 | 0.223 | 0.136 |
| 11 | | 4.104 | 1.796 | 2.285 | 0.010 | 0.365 | 0.027 |
| 12 | | 6.306 | 0.991 | 6.357 | 0.074 | 0.133 | 0.558 |
| Total | Mean | 4.752 | 1.084 | 4.782 | 0.035 | 0.228 | 0.313 |
|  | SD | 1.953 | 0.308 | 2.425 | 0.037 | 0.101 | 0.421 |

**Table S16. Detrended temporal stability of simulated aboveground biomass and forest productivity during early development period (i.e., 1~50 simulation years).**

| **Plot** | | **Aboveground Biomass** | | | **Forest Productivity** | | |
| --- | --- | --- | --- | --- | --- | --- | --- |
|  |  | **Mean** | **SD(Residual)** | **Stability** | **Mean** | **SD(Residual)** | **Stability** |
| 1 | | 5.193 | 0.249 | 20.835 | 0.142 | 0.108 | 1.316 |
| 2 | | 5.042 | 0.280 | 18.012 | 0.146 | 0.105 | 1.382 |
| 3 | | 3.960 | 0.487 | 8.130 | 0.072 | 0.206 | 0.352 |
| 4 | | 3.474 | 0.772 | 4.499 | 0.046 | 0.345 | 0.134 |
| 5 | | 5.194 | 0.228 | 22.783 | 0.160 | 0.076 | 2.105 |
| 6 | | 3.368 | 0.630 | 5.347 | 0.087 | 0.253 | 0.343 |
| 7 | | 3.436 | 0.720 | 4.775 | 0.064 | 0.272 | 0.234 |
| 8 | | 2.587 | 0.381 | 6.797 | 0.066 | 0.196 | 0.336 |
| 9 | | 2.248 | 0.357 | 6.303 | 0.053 | 0.177 | 0.298 |
| 10 | | 5.472 | 0.222 | 24.623 | 0.102 | 0.159 | 0.640 |
| 11 | | 4.096 | 0.585 | 7.000 | 0.096 | 0.228 | 0.422 |
| 12 | | 4.444 | 0.180 | 24.673 | 0.139 | 0.130 | 1.061 |
| Total | Mean | 4.043 | 0.424 | 12.815 | 0.124 | 0.188 | 0.719 |
|  | SD | 1.059 | 0.208 | 8.500 | 0.098 | 0.079 | 0.611 |

**Table S17. Detrended temporal stability of simulated aboveground biomass and forest productivity during late development period (i.e., 51~97 simulation years).**

| **Plot** | | **Aboveground Biomass** | | | **Forest Productivity** | | |
| --- | --- | --- | --- | --- | --- | --- | --- |
|  |  | **Mean** | **SD(Residual)** | **Stability** | **Mean** | **SD(Residual)** | **Stability** |
| 1 | | 9.148 | 0.358 | 25.590 | 0.015 | 0.093 | 0.160 |
| 2 | | 9.276 | 0.370 | 25.038 | 0.022 | 0.091 | 0.236 |
| 3 | | 3.663 | 0.883 | 4.149 | **–**0.073 | 0.369 | **–**0.197 |
| 4 | | 2.578 | 0.548 | 4.703 | **–**0.044 | 0.270 | **–**0.161 |
| 5 | | 10.085 | 0.354 | 28.481 | 0.027 | 0.086 | 0.312 |
| 6 | | 3.579 | 0.865 | 4.136 | **–**0.069 | 0.416 | **–**0.165 |
| 7 | | 2.947 | 0.853 | 3.454 | **–**0.059 | 0.349 | **–**0.171 |
| 8 | | 2.789 | 0.783 | 3.562 | **–**0.050 | 0.321 | **–**0.155 |
| 9 | | 2.399 | 0.553 | 4.336 | **–**0.032 | 0.244 | **–**0.131 |
| 10 | | 7.006 | 0.639 | 10.961 | **–**0.051 | 0.277 | **–**0.183 |
| 11 | | 4.075 | 1.318 | 3.093 | **–**0.088 | 0.472 | **–**0.188 |
| 12 | | 8.262 | 0.317 | 26.099 | 0.003 | 0.136 | 0.023 |
| Total | Mean | 5.484 | 0.653 | 11.967 | **–**0.033 | 0.260 | **–**0.052 |
|  | SD | 3.009 | 0.300 | 10.809 | 0.040 | 0.133 | 0.185 |

## References

Chave, J., Coomes, D., Jansen, S., Lewis, S. L., Swenson, N. G., & Zanne, A. E. (2009). Towards a worldwide wood economics spectrum. *Ecology Letters, 12*(4), 351-366. doi:10.1111/j.1461-0248.2009.01285.x

Dietze, M. C., Lebauer, D. S., & Kooper, R. (2013). On improving the communication between models and data. *Plant, Cell and Environment, 36*(9), 1575-1585. doi:10.1111/pce.12043

Dong, T. (2015). *Effects of artificial regulation on growth in Cunninghamia lanceolata.* University of Chinese Academy of Sciences, Beijing.

Dong, T., Zhang, Y., Zhang, Y., & Zhang, S. (2015). Continuous planting under a high density enhances the competition for nutrients among young Cunninghamia lanceolata saplings. *Annals of Forest Science, 73*(2), 331-339. doi:10.1007/s13595-015-0518-1

Feng, X., Uriarte, M., Gonzalez, G., Reed, S., Thompson, J., Zimmerman, J. K., & Murphy, L. (2018). Improving predictions of tropical forest response to climate change through integration of field studies and ecosystem modeling. *Global Change Biology, 24*, e213-e232. doi:10.1111/gcb.13863

Gompertz, B. (1997). On the nature of the function expressive of the law of human mortality, and on a new mode of determining the value of life contingencies. In a letter to Francis Baily, Esq. F. R. S. &c. By Benjamin Gompertz, Esq. F. R. S. Abstracts of the Papers Printed in the Philosophical Transactions of the Royal Society of London, 2, 252-253.

Jucker, T., Fischer, F. J., Chave, J., Coomes, D. A., Caspersen, J., Ali, A., . . . Zavala, M. A. (2022). Tallo: A global tree allometry and crown architecture database. *Global Change Biology, 28*(17), 5254-5268. doi:10.1111/gcb.16302

Kattge, J., Bonisch, G., Diaz, S., Lavorel, S., Prentice, I. C., Leadley, P., . . . Wirth, C. (2020). TRY plant trait database - enhanced coverage and open access. *Global Change Biology, 26*(1), 119-188. doi:10.1111/gcb.14904

Kiviste, A. K. (1988). Mathematical functions of forest growth. Estonian Agricultural Academy, Tartu, USSR.

LeBauer, D., Kooper, R., Mulrooney, P., Rohde, S., Wang, D., Long, S. P., & Dietze, M. C. (2018). BETYdb: a yield, trait, and ecosystem service database applied to second‐generation bioenergy feedstock production. *GCB Bioenergy, 10*(1), 61-71. doi:10.1111/gcbb.12420

LeBauer, D. S., Wang, D., Richter, K. T., Davidson, C. C., & Dietze, M. C. (2013). Facilitating feedbacks between field measurements and ecosystem models. *Ecological Monographs, 83*(2), 133-154. doi:10.1890/12-0137.1

Li, K., & Xiang, W.-h. (2011). Comparison of specific leaf area, SPAD value and seed mass among subtropical tree species in hilly area of Central Hunan, China. *Journal of Central South University of Forestry & Technology, 31*(5), 213-218.

Li, X.-R., Liu, Q.-i. J., Cai, Z., & Ma, Z.-Q. (2007). Specific leaf area and leaf area index of conifer plantations in Qianyanzhou station of subtropical China. *Journal of Plant Ecology (Chinese Version), 31*(1), 93-101.

Li, Y. (2016). *Ecophysiological responses of a subtropical coniferous-broadleaved mixed forest to climate warming.* (Doctor), University of Chinese Academy of Sciences, Beijing.

Li, Y. (2019). *Study on the relationship between maximum carboxylation rate and leaf biochemical components and the simulation of photosynthesis in tropical/subtropical evergreen forests.* (Doctor), University of Chinese Academy of Sciences Beijing.

Li, Y., Zhou, L., Wang, S., Chi, Y., & Chen, J. (2018). Leaf temperature and vapour pressure deficit (VPD) driving stomatal conductance and biochemical processes of leaf photosynthetic rate in a subtropical evergreen coniferous plantation. *Sustainability, 10*(11). doi:10.3390/su10114063

Mitscherlich, E. A. (1909). Das gesetz des minimums und das gesetz des abnehmenden bodenertrages (in German). *Landwirtsch Jahrbuch, 38*, 537-552.

Paine, C. E. T., Amissah, L., Auge, H., Baraloto, C., Baruffol, M., Bourland, N., . . . Gibson, D. (2015). Globally, functional traits are weak predictors of juvenile tree growth, and we do not know why. *Journal of Ecology, 103*(4), 978-989. doi:10.1111/1365-2745.12401

Prentice, I. C., Meng, T., Wang, H., Harrison, S. P., Ni, J., & Wang, G. (2011). Evidence of a universal scaling relationship for leaf CO2 drawdown along an aridity gradient. *New Phytologist, 190*(1), 169-180. doi:10.1111/j.1469-8137.2010.03579.x

R_Core_Team. (2021). R: A language and environment for statistical computing. *R Foundation for Statistical Computing.* [*https://www.R-project.org/*](https://www.R-project.org/).

Richards, F. J. (1959). Aflexible growth function for empirical use. *Journal of Experimental Botany, 29*, 290-300.

Shiklomanov, A. N., Dietze, M. C., Fer, I., Viskari, T., & Serbin, S. P. (2021). Cutting out the middleman: calibrating and validating a dynamic vegetation model (ED2-PROSPECT5) using remotely sensed surface reflectance. *Geoscientific Model Development, 14*(5), 2603-2633. doi:10.5194/gmd-14-2603-2021

Verhulst, P. F. (1838). Notice sur la loi que la populaions suit dans son accroissement (in French). *Correspondance Mathématique et Physique, 10*, 113-121.

Wang, C., Liu, S., Zhu, Y., Smith, A. R., Ning, Y., & Deng, D. (2024). Aboveground carbon sequestration of Cunninghamia lanceolata forests: Magnitude and drivers. *Forest Ecosystems, 11*, 100165. doi:10.1016/j.fecs.2024.100165

Wang, H., Harrison, S. P., Li, M., Prentice, I. C., Qiao, S., Wang, R., . . . Yang, Y. (2022). The China plant trait database version 2. *Scientific Data, 9*(1), 769. doi:10.1038/s41597-022-01884-4

Wang, H., Harrison, S. P., Prentice, I. C., Yang, Y., Bai, F., Togashi, H. F., . . . Ni, J. (2018). The China Plant Trait Database: toward a comprehensive regional compilation of functional traits for land plants. *Ecology, 99*(2), 500-500. doi:<https://doi.org/10.1002/ecy.2091>

Wang, Y. (2010). *Simulation about the carbon and water cycle based on EPPML.* (Master), Graduate University of Chinese Academy of Sciences, Beiing.

Weibull, W. (1951). A statistical distribution function of wide applicability. *Journal of Applied Mechanics, American Society of Mechanical Engineers, 18*(3), 293-297.

Wu, Q., Song, C., Song, J., Wang, J., Chen, S., Yang, L., . . . Jiang, J. (2021). Effects of leaf age and canopy structure on gross ecosystem production in a subtropical evergreen Chinese fir forest. *Agricultural and Forest Meteorology, 310*. doi:10.1016/j.agrformet.2021.108618

Xie, M., Zhu, Y., Liu, S., Deng, D., Zhu, L., Zhao, M., & Wang, Z. (2022). Simulating the Impacts of Drought and Warming in Summer and Autumn on the Productivity of Subtropical Coniferous Forests. *Forests, 13*, 2147. doi:10.3390/f13122147

Xu, H., Wang, H., Prentice, I. C., Harrison, S. P., Wang, G., & Sun, X. (2021). Predictability of leaf traits with climate and elevation: a case study in Gongga Mountain, China. *Tree Physiology, 41*(8), 1336-1352. doi:10.1093/treephys/tpab003

Xu, H., Wang, H., Prentice, I. C., Harrison, S. P., & Wright, I. J. (2021). Coordination of plant hydraulic and photosynthetic traits: confronting optimality theory with field measurements. *New Phytologist, 232*(3), 1286-1296. doi:10.1111/nph.17656

Ye, W. (2019). *Effects of simulated warming on photosynthetic characteristics and product distribution of Cunninghamia lanceolata.* (Master), Fujian Normal University, Fuzhou.

Zhao, M., Xiang, W., Deng, X., Tian, D., Huang, Z., Zhou, X., . . . Peng, C. (2013). Application of TRIPLEX model for predicting Cunninghamia lanceolata and Pinus massoniana forest stand production in Hunan Province, southern China. *Ecological Modelling, 250*, 58-71. doi:10.1016/j.ecolmodel.2012.10.011

Zhou, L. (2013). *The effect of drought on the carbon cycle of China's terrestrial ecosystems during 2001-2010.* (Doctor of Science), The University of Chinese Academy of Sciences, Beijing.

Zhu, Y., Liu, S., Yan, W., Deng, D., Zhou, G., Zhao, M., . . . Xie, M. (2022). Impact of Ice-Storms and Subsequent Salvage Logging on the Productivity of Cunninghamia lanceolata (Chinese Fir) Forests. *Forests, 13*(2), 296. doi:10.3390/f13020296
